# Causal relationship between immune cells and periodontitis: A Mendelian randomization study

**DOI:** 10.1097/MD.0000000000040918

**Published:** 2024-12-13

**Authors:** Junlei Bi, Yuxin Chen, Jie Zhang, Jiaqi Yan, Aiyun Ge, Wenhao Ye, Changqing Liu, Hebao Wen, Caiyun Ma

**Affiliations:** a Anhui Engineering Research Center for Neural Regeneration Technology and Medical New Materials, School of Life Science, Bengbu Medical University, Bengbu City, Anhui Province, China; b Physical Education Department, Bengbu Medical University, Bengbu City, Anhui Province, China.

**Keywords:** causal relationships, immune cells, Mendelian randomization, periodontitis

## Abstract

This study employed Mendelian randomization (MR) analysis to explore potential causal relationships between 731 immune cell subtypes and periodontitis. Utilizing a 2-sample MR design, our study delved into the diverse landscape of immune cell interactions with periodontitis-associated factors. Multiple MR methods, including inverse variance weighting, weighted median, and MR-Egger tests, were employed to ensure reliability and mitigate potential pleiotropic effects. The study revealed significant causal effects (FDR < 0.15) between immune cells (B cells, maturation stages of T cells, Treg) and periodontitis. Notably, receptors like triggering receptor expressed on myeloid cells-1 (TREM-1) and triggering receptor expressed on myeloid cells-2 (TREM-2) exhibited intricate roles, warranting further investigation. In conclusion, this MR analysis elucidates complex causal relationships between immune cell subtypes and periodontitis. The findings provide a foundation for understanding systemic implications, offering insights for clinical practice and highlighting avenues for future research.

## 1. Introduction

Periodontitis is a multifactorial inflammatory disease that results in the loss of supporting tissues around the teeth, contributing to a systemic inflammatory burden. Severe periodontitis affects approximately 10% of the global population.^[1]^ This underscores its significance, providing sustained impetus for relevant research. The severity and extent of periodontitis depend on the interplay between triggering microbial factors and the host immune system.^[2]^

Relevant research suggests that within tissues impacted by periodontitis, there is a markedly elevated abundance of intermediate monocytes and nonclassical monocytes. Intermediate monocytes represent the most pro-inflammatory subtype within the monocyte population, and their perturbation has been documented in various inflammatory and autoimmune diseases.^[3–5]^ Recent studies have concurrently indicated that the primary secretory factor of interleukin-17 (Th17), interleukin-17 (IL-17), plays a dual role in the process of alveolar bone resorption. IL-17 cells are considered early responders in the immune defense against infection, playing a crucial role in the innate immune system. Throughout the process of bone destruction, IL-17 can induce fibroblasts, epithelial cells, and endothelial cells to produce a plethora of inflammatory cytokines. These cytokines include IL-6, IL-8, monocyte chemoattractant protein-1, granulocyte chemotactic protein-2, human β-defensin-2, macrophage inflammatory protein-3, tumor necrosis factor-α (TNF-α), and granulocyte-macrophage colony-stimulating factor, among others. This induction facilitates the recruitment of numerous neutrophils and macrophages to the site of tissue infection, aiming to clear bacteria and infectious substances, mediate the host’s immune response against periodontal tissue inflammation, ultimately resulting in alveolar bone resorption. Moreover, the chemotaxis accuracy of neutrophils in the peripheral blood of periodontitis patients is compromised, potentially prolonging their tissue transit time and consequently leading to collateral tissue damage.^[6]^ Furthermore, there exists a reciprocal interaction between periodontitis and overall health and diseases. Global health organizations have recognized the oral health status as a key determinant of human health. To date, more than 50 diseases have been associated with periodontitis, including cardiovascular diseases,^[7]^ Alzheimer disease^[8]^ and inflammatory bowel disease (IBD).^[9]^ Among them, IBD has garnered particular attention.^[10,11]^

Mendelian randomization (MR) utilizes genetic variants strongly associated with both the exposure factor (immune cells) and the outcome (periodontitis) as instrumental variables (IVs). This approach aims to infer the causal effects between immune cells and periodontal disease, leveraging the robust genetic correlation between the exposure and outcome variables.^[12]^ At present, traditional experimental studies are constrained by factors such as limited experimental funding, time constraints, and other objective limitations, resulting in relatively small sample sizes at the individual-level. This limitation hinders a comprehensive elucidation of the causal relationship between exposure factors and outcomes. However, MR analysis based on aggregate statistics is approximately equivalent to individual-level MR analysis. Two-sample MR analysis employs single nucleotide polymorphisms (SNPs) from independent genome-wide association studies (GWAS) for exposure and outcome associations, combining them to derive a unified causal inference.

Hence, this study conducted a comprehensive 2-sample MR analysis to ascertain the causal relationship between immune cell characteristics and periodontitis. This has significant implications for clinical treatment strategies and the enhancement of overall quality of life for the population.

## 2. Materials and methods

### 2.1. Study design

**Figure 1.**
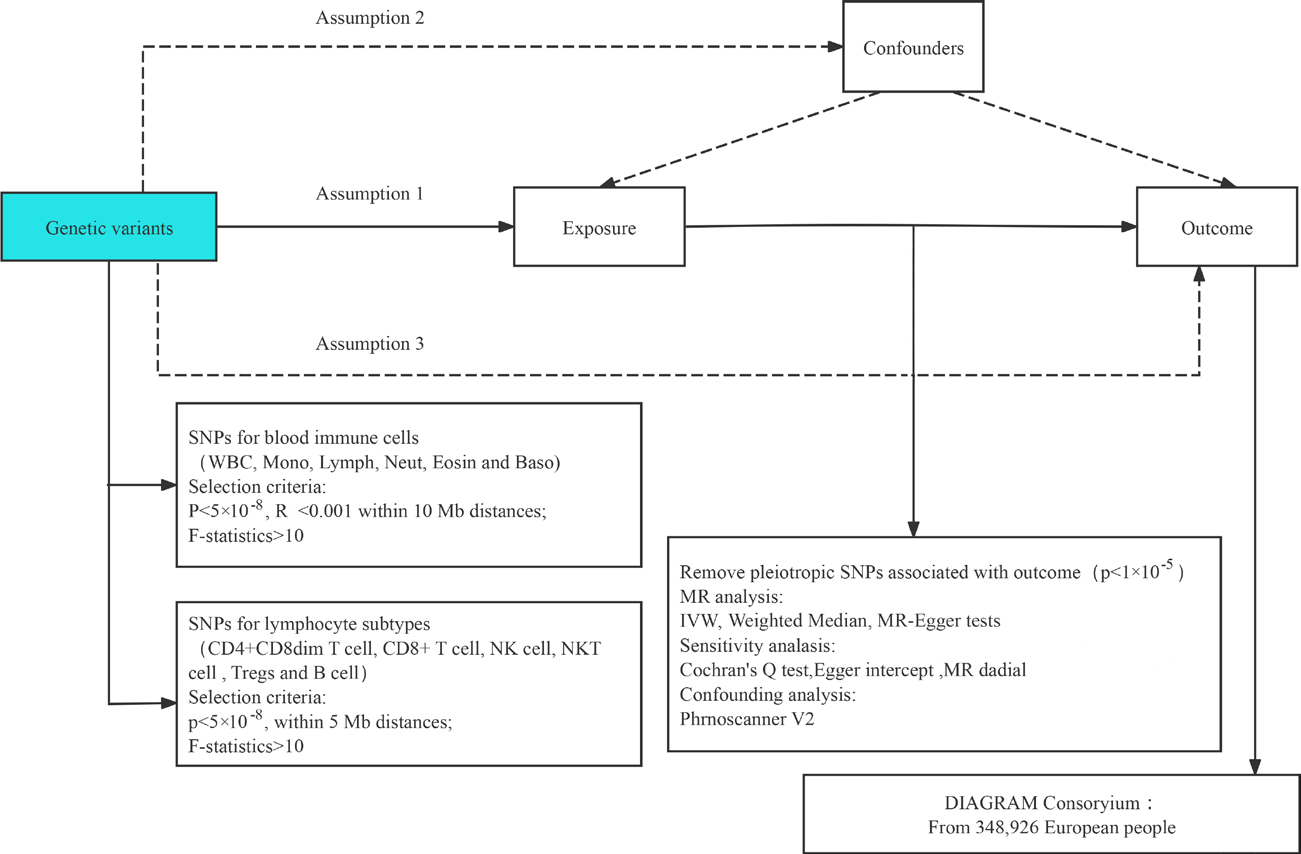
Overview of the overall MR design: assumption 1, instrumental variables strongly correlated with confounding factors; assumption 2, instrumental variables uncorrelated with confounding factors; assumption 3, instrumental variables exclusively associated with exposure and outcome. IVW = inverse variance weighting, LD = linkage disequilibrium, LOO = leave-one-out, MR = Mendelian randomization, SNP = single nucleotide polymorphism.

We employed a 2-sample MR analysis to assess the causal relationship between 731 immune cell characteristics and periodontitis. In MR, genetic variants serve as proxies for risk factors; therefore, IVs in causal inference must adhere to 3 core assumptions: the relevance assumption, where SNPs are strongly correlated with the exposure factor; the independence assumption, asserting independence between SNPs and confounding factors; and the exclusion restriction assumption, stipulating that SNPs only influence the outcome through the exposure factor. The overall technical roadmap is illustrated in Figure 1.

### 2.2. The data source for the GWAS on periodontitis

The summary statistics for GWAS on periodontitis were obtained from the IEU Open GWAS project (https://gwas.mrcieu.ac.uk) with GWAS ID ebi-a-GCST90018897. This study conducted a GWAS on 348,926 European individuals (N case = 1740, N control = 347,186), analyzing approximately 9.5 million variants after quality control and imputation. The GWAS identified 128 independent SNPs, including 83 not previously reported, with at least 108 located at independent genomic loci reaching genome-wide significance (*P* < 5 × 10^−8^).

### 2.3. The immunome-wide GWAS data source

We downloaded the summary statistics for a total of 731 immune traits from the GWAS catalog (accession numbers GCST0001391 to GCST0002121). These encompassed 731 immune phenotypes, including absolute cell (AC) counts (n = 118), median fluorescence intensity (MFI) reflecting surface antigen levels (n = 389), morphological parameters (MP) (n = 32), and relative cell (RC) counts (n = 192). Specifically, MFI, AC, and RC functionalities encompassed B cells, CDC, mature T cells, monocytes, myeloid cells, TBNK (T cells, B cells, natural killer cells), and regulatory T cell groups, while MP functionalities included CDC and TBNK groups. The initial GWAS was conducted using data from 3757 European individuals without overlapping cohorts. Imputation of approximately 22 million SNPs, based on the Sardinian sequence as a reference panel, was performed using genotyping data from a high-density array. Covariates such as gender, age, and age squared were adjusted during analysis.

### 2.4. Selection of IVs

According to recent research,^[13,14]^ the significance threshold for each IV for immune traits was set at 1 × 10^−5^. The clustering process version (v1.90) in PLINK software was employed to prune these SNPs (linkage disequilibrium [LD] *r*^2^ threshold < 0.001 within 10,000 kb distance), where LD *r*^2^ was computed based on the 1000 Genomes Project as a reference panel. False discovery rate (FDR) correction was conducted by applied q-value procedure, when FDR *q* < 0.15 was considered statistically significant, *P* < .05 but *q* ≧ 0.1 were considered to have a suggestive association.^[15]^

For periodontitis, the significance level was adjusted to 5 × 10^−8^ and *R*^2^ <0.001 within 10 Mb distances. Phenotypic variance explained and *F*-statistics were computed for each IV to assess instrument strength and mitigate weak instrument bias. A total of 7 to 1786 independent immune phenotype IVs were identified, explaining an average of 0.240% (range 0.004%–3.652%) of variance for their respective immune traits. Subsequently, after removing IVs with low *F*-statistics (<10), 110 IVs for periodontitis were retained for further analysis.

Power and instrumental strength requirements for our MR study by using multiple genetic variants, and if the robustness of the IVs was verified by calculating the *F*-statistic using the formula *F* = (beta^2^)/se^2^ where *F* < 10 indicates a possible weak IV.

### 2.5. Statistical analysis

We used several methods, primarily the inverse variance weighting (IVW) method, with the weighted median method and the MR-Egger method as secondary methods. MR results are expressed as β and its corresponding 95% confidence interval (CI). Differences were statistically significant when IVW *P* < .05 and the other 2 methods were in the same direction.

All analyses were conducted using R 4.3.1 software (http://www.Rproject.org). To assess the causal relationship between 731 immune phenotypes and periodontitis, we primarily employed the “MR” package (version 0.4.3)^[16]^ for IVW,^[17]^ weighted median,^[18]^ and MR-Egger tests^.[19]^ Heterogeneity among selected IVs was evaluated using Cochran *Q* statistic and the corresponding *P*-value. If the null hypothesis was rejected, a random-effects IVW was used instead of the fixed-effects IVW. To account for horizontal pleiotropy, a common method, MR-Egger, was employed, where a significant intercept term indicates the presence of horizontal pleiotropy.^[20]^ Additionally, a robust method, MR-Straw and outlier (MR-STRO), was used to exclude potential horizontal pleiotropy outliers that could significantly impact estimates in the MR-STRO package.^[21]^ Scatterplots and funnel plots were also utilized. Scatterplots illustrated that results were not influenced by outliers, and funnel plots demonstrated the robustness and absence of heterogeneity in the correlation.

## 3. Results

### 3.1. Exploration of the pathogenic role of immune cells in periodontitis

After FDR adjustment (FDR < 0.15), we investigated the pathogenic relevance of immune phenotypes to periodontitis: B cells, maturation stages of T cells, and Treg. Specifically, using the IVW method, the initial estimation of the odds ratio (OR) for CD39 + activated CD4 regulatory T cell was 0.959 (95% CI = 0.933–0.985, *P* = 2.350 × 10^−3^, FDR = 0.112) (Supplementary File 1, Supplemental Digital Content, http://links.lww.com/MD/O164). Similarly, For CD4 on effector memory CD4 + T cell in relation to the risk of periodontitis was 1.169 (95% CI = 1.056–1.295, *P* = 2.735 × 10^−3^, FDR = 0.112). The IVW method estimated an OR of 0.964 (95% CI = 0.940–0.987, *P* = 2.740 × 10^−3^, FDR = 0.112) for CD39 + secreting CD4 regulatory T cell in relation to the risk of periodontitis. OR for CD27 on switched memory B cell in relation to the risk of periodontitis, using the IVW method, was 1.066 (95% CI = 1.023–1.110, *P* = 2.316 × 10^−3^, FDR = 0.112). The IVW method estimated an OR of 0.960 (95% CI = 0.935–0.986, *P* = 2.564 × 10^−3^, FDR = 0.112) for CD39 + CD8 + T cell in relation to the risk of periodontitis. The estimated OR for CD39 + CD4 + T cell in relation to the risk of periodontitis was 0.963 (95% CI = 0.939–0.987, *P* = 2.411 × 10^−3^, FDR = 0.112).

**Figure 2.**
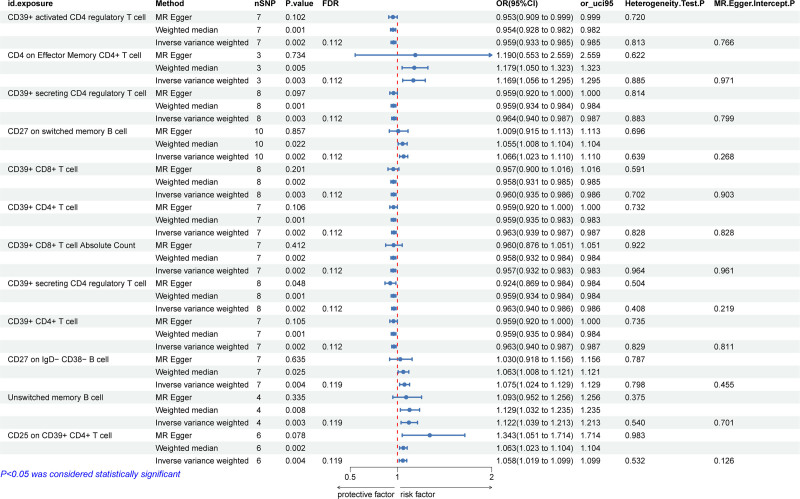
Forest plot illustrating the causal relationship between periodontitis and immune phenotypes. CI = confidence interval, IVW = inverse variance weighting.

OR for CD39 + CD8 + T cell absolute count in relation to the risk of periodontitis was 0.957 (95% CI = 0.932–0.983, *P* = 1.507 × 10^−3^, FDR = 0.112), the risk of CD39 + secreting CD4 regulatory T cell was 0.963 (95% CI = 0.940–0.986, *P* = 1.504 × 10^−3^, FDR = 0.112), OR of CD39 + CD4 + T cell risk was estimated to be 0.963 (95% CI = 0.940–0.986, *P* = 2.397 × 10^−3^, FDR = 0.112, OR of CD27 on IgD- CD38- B cell risk was estimated to be 1.075 (95% CI = 1.024–1.129, *P* = 3.575 × 10^−3^, FDR = 0.119), the estimated OR for Unswitched memory B cell in relation to the risk of periodontitis was 1.122 (95% CI = 1.039–1.213, *P* = 3.380 × 10^−3^, FDR = 0.119), OR of CD25 on CD39 + CD4 + T cell risk was estimated to be 1.058 (95% CI = 1.019–1.099, *P* = 3.697 × 10^−3^, FDR = 0.119 (Fig. 2). In addition, both MR-Egger intercept and the global test for MRP 10 ruled out the possibility of horizontal pleiotropy in these 4 associations. Detailed information from sensitivity analysis demonstrates the robustness of the observed causal relationships (Supplementary Files 2 and 3, Supplemental Digital Content, http://links.lww.com/MD/O164). Scatter plots and funnel plots also indicate the stability of the results (Supplementary File 4, Supplemental Digital Content, http://links.lww.com/MD/O165).

## 4. Discussion

The current study employs MR analysis, widely utilized to elucidate potential causal relationships between risk factors and outcomes. Utilizing this 2-sample MR framework, we investigated causal relationships between 731 immune cell phenotypes and factors influencing the treatment of periodontitis. To ensure the reliability of the MR analysis and mitigate potential pleiotropic effects, we employed various MR methods, including weighted median, and MR-Egger tests. Notably, this study represents the first exploration of MR analysis between immune cell phenotypes and pathogenic factors of periodontitis, identifying B cell, Maturation stages of T cells and Treg with significant causal effects on periodontitis (FDR < 0.15).

The research findings indicate a causal relationship between Maturation stages of T cells and periodontitis. CD39 expression is associated with myeloid cells. A review of relevant literature reveals that the occurrence, development, and periodontal tissue damage in periodontitis are primarily associated with the host’s immune response to various bacteria and their byproducts within the dental plaque biofilm.^[22]^ Myeloid cells triggering receptors belong to the immunoglobulin superfamily.^[23]^ They are expressed not only on the cell surface of late-stage differentiated bone marrow cells such as monocytes, macrophages, and neutrophils but also in epithelial cells, connective tissues, and tumor tissues.^[24,25]^ TREMs include activating and inhibitory subtypes encoded by the major histocompatibility complex and play a crucial role in the immune system.^[26–28]^ Among them, TREM-1 and TREM-2 activate myeloid cells through the adapter protein DAP12 signaling.^[29]^

The triggering receptor expressed on TREM-1, expressed on myeloid cells, including monocytes, macrophages, and neutrophils, is a crucial modulator in the innate immune response to bacterial infections, as evidenced by numerous experiments.^[30–32]^ It triggers the secretion of pro-inflammatory chemokines and cytokines by phagocytes, amplifying the inflammatory response induced by bacteria and fungi.^[33,34]^ The synergistic action between TREM-1 and Toll-like receptors results in an amplification loop activating the NF-κB pathway.^[35]^ This loop contributes to the increased production of pro-inflammatory cytokines such as IL-1β and TNF-α and the inhibition of IL-10 production.^[36]^

Previous studies have demonstrated elevated concentrations of TREM-1 in saliva and gingival crevicular fluid from individuals with periodontitis.^[37]^ The presence of TREM-1 has also been detected in gingival tissues of patients with periodontitis, correlating with the presence of red complex bacteria.^[38]^ Analysis of gingival crevicular fluid and subgingival plaque samples from healthy individuals, as well as those with chronic or aggressive periodontitis, revealed a significant elevation in the secretion levels of soluble TREM-1 (sTREM-1) in the periodontitis groups compared to the healthy group. This supports the notion that the expression levels of sTREM-1 can reflect the extent of periodontal disease pathology to some extent.

As a negative regulatory factor in the inflammatory response, TREM-2 can play a role in chronic inflammation by stimulating the production of chemokines and cytokines that constitute, rather than induce, inflammation.^[39]^ However, its specific role in periodontitis requires further investigation. Recently, TREM-2 expression is not detected in the gingival epithelium of normal individuals. Additionally, TREM-2 forms a receptor signaling complex with transmembrane immune signaling adaptor (TYROBP) to activate the immune response of macrophages and dendritic cells.^[40]^ It activates dendritic cells derived from monocytes and serves as a phagocytic receptor to recognize and bind to certain bacteria and fungi, enhancing microbial clearance to strengthen the host’s immune response^.[41]^ This contrasts with the mechanism of our inflammatory response, suggesting that the causal relationship between TREM-2 and periodontitis warrants further investigation.

Furthermore, the data results indicate the association between the occurrence and progression of periodontitis and the majority of Treg. Treg are a subset of T cells that secrete IL-10 and TGF-β, playing a role in suppressing immune responses.^[42]^ IL-10, primarily derived from monocytes and macrophages during inflammatory reactions, mitigates periodontal tissue destruction by inhibiting RANKL and matrix metalloproteinases.^[43]^ TGF-β, negatively correlated with the expression of nuclear factor κB ligand receptor, possesses certain chemotactic properties. It induces the aggregation of immune cells, including neutrophils and macrophages, at infection sites, leading to the release of IL-1, IL-6, and TNF-α, promoting inflammation. Additionally, TGF-β can stimulate the differentiation of helper T cells 17, inducing an increase in IL-17, facilitating the formation of osteoclastogenic mediators, and exacerbating the progression of periodontitis.^[44]^ Thus, whether in immune tolerance to periodontitis or related inflammatory responses, Treg plays a crucial role.

Numerous experimental results reveal that the proportion of Treg in peripheral blood is significantly higher in patients with both periodontitis and cancer compared to non-cancer patients or cancer patients without periodontitis.^[45]^ Given that periodontitis is a systemic reactive and chronic inflammatory disease, systemic Treg levels are markedly elevated, contributing to immune evasion in cancer.^[46]^ Based on these findings, Treg may inhibit relevant anticancer immune responses, affecting the immune system’s surveillance of tumors, ultimately leading to tumor development. Therefore, Treg is of significant importance in both the occurrence and progression of periodontitis and the emergence of human tumors. In clinical practice, monitoring Treg levels can be used to assess the severity of periodontitis and tumor development.

It is noteworthy that B cells play a crucial role in regulating immune responses, inducing tissue destruction, and eliminating periodontal pathogens. Extensive transcriptomic and histological evidence indicates that B cell infiltration plays a vital role in host defense against periodontitis. Within the oral periodontal system, specific subtypes of B cells have been shown to correlate with disease severity, and the B cell-stimulating cytokines BLyS (B lymphocyte stimulator) and APRIL (a proliferation-inducing ligand) have been demonstrated to elevate in periodontitis.^[47,48]^ Therefore, these cells and cytokines may regulate osteoclastogenesis, playing a key role in the progression of periodontitis.

The advantage of this article is that MR studies can use existing large-scale GWAS data, which can be used to study the causal relationship between immune cells and periodontitis faster and faster. At the same time, the use of genetic variation in GWAS analysis as IVs can help overcome the bias inherent in observational studies, such as confounding factors and reverse causal bias, thereby strengthening the causal link between exposure factors and outcome variables. However, this study has certain limitations. The relatively lenient thresholds used in the experimental data may lead to some false-positive results. Additionally, the data source for this experiment is from the European population, and the sample size is not large enough. Considering racial differences, the results of this experimental data may exhibit variations in comparison to the Asian population. These issues need to be better addressed in future in-depth studies.

## 5. Conclusion

To our knowledge, this is the first study to explore the causal relationship between immune cells and periodontitis by using a 2-sample MR analysis method. Our research provide a useful direction for the next experimental studies to explore the mechanisms underlying the interactions between periodontitis and immune cells. We believe that our results will provide new insights in the immunotherapy for periodontitis disease.

## Acknowledgments

The authors appreciate the original study investigators for sharing their valuable information.

## Author contributions

**Conceptualization:** Junlei Bi, Yuxin Chen.

**Formal analysis:** Aiyun Ge, Wenhao Ye.

**Funding acquisition:** Hebao Wen, Caiyun Ma.

**Investigation:** Aiyun Ge, Wenhao Ye.

**Methodology:** Jie Zhang, Jiaqi Yan.

**Resources:** Hebao Wen, Caiyun Ma.

**Supervision:** Hebao Wen, Caiyun Ma.

**Writing – original draft:** Junlei Bi.

**Writing – review & editing:** Changqing Liu, Hebao Wen, Caiyun Ma.

## Supplementary Material


