# Supplementary material for: Causal relationship between immune cells and periodontitis: A Mendelian randomization study
Source: Medicine (Baltimore). 2024 Dec 13;103(50):e40918. doi: 10.1097/MD.0000000000040918 (PMC11651513; doi:10.1097/MD.0000000000040918)
Supplement: Supplementary file 2 [file medi-103-e40918-s002.docx]

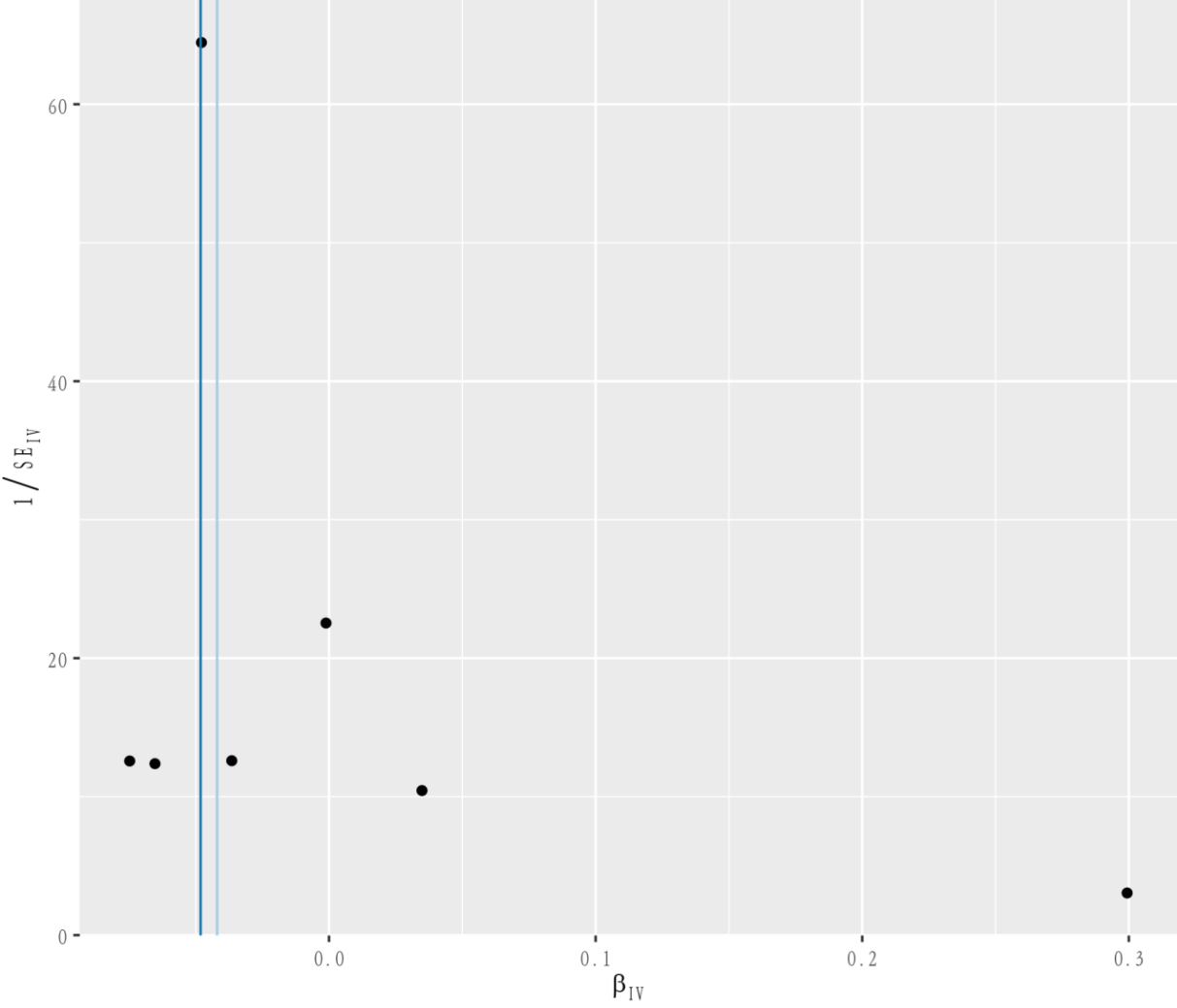


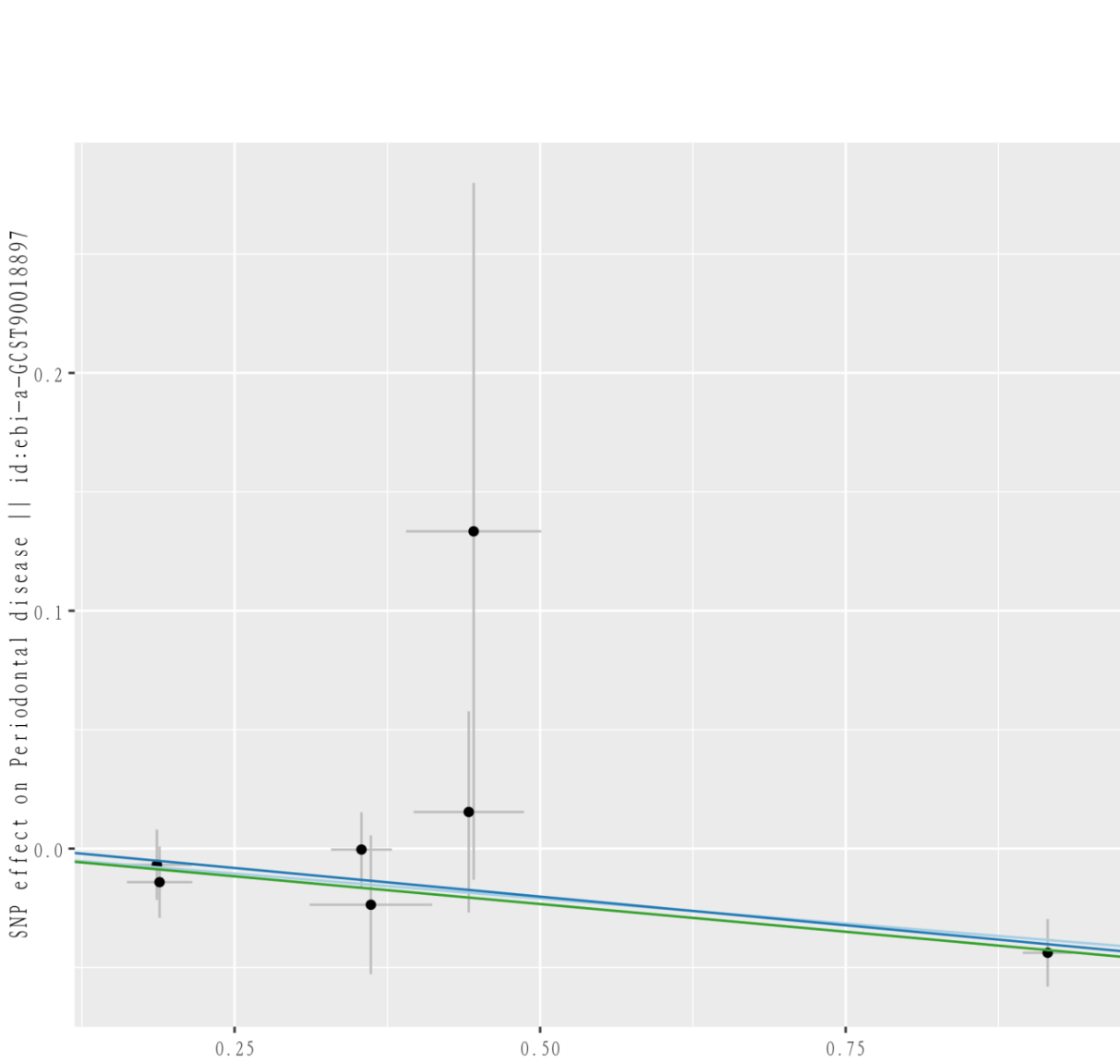


CD39+ activated CD4 regulatory T cell


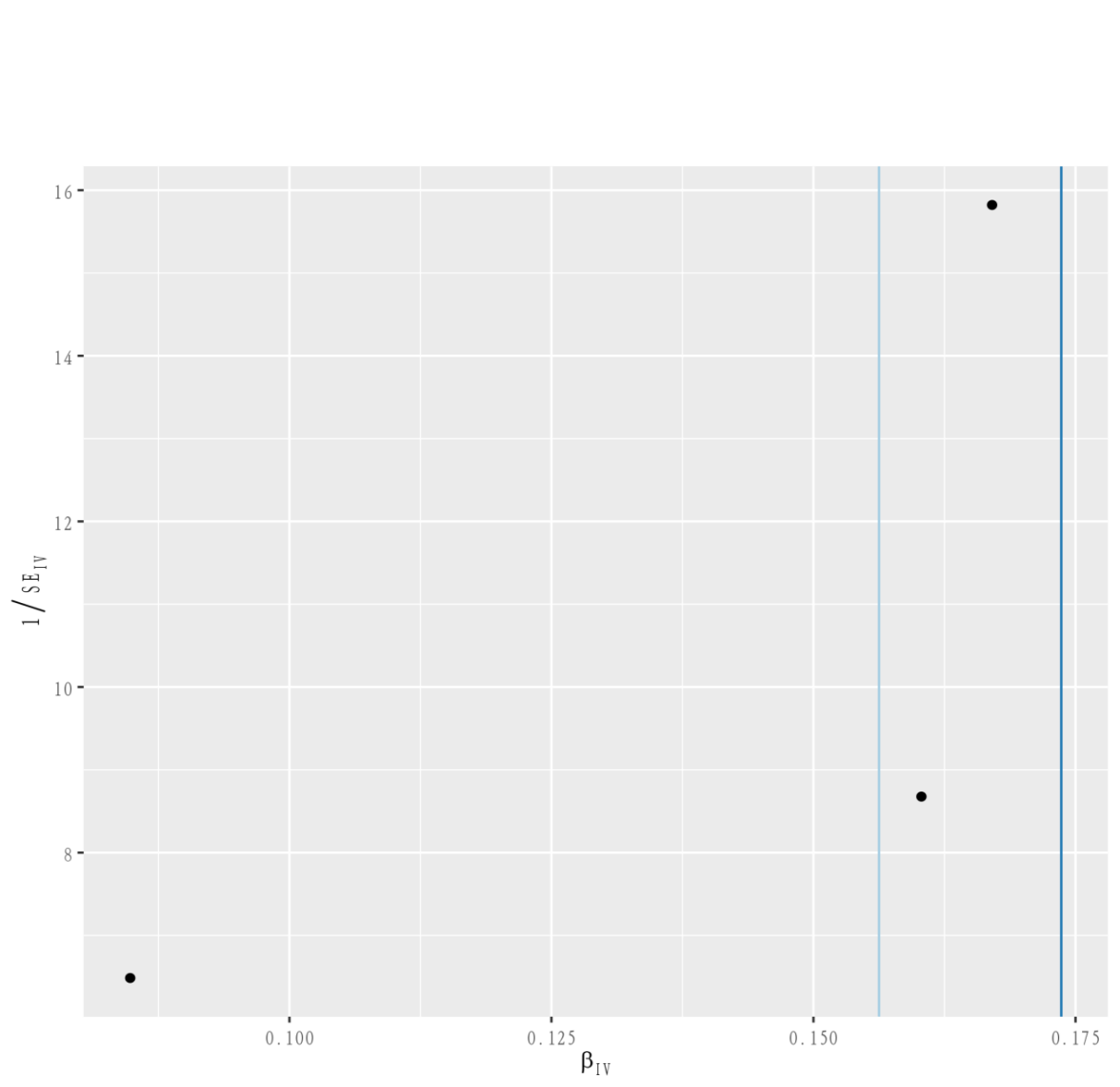


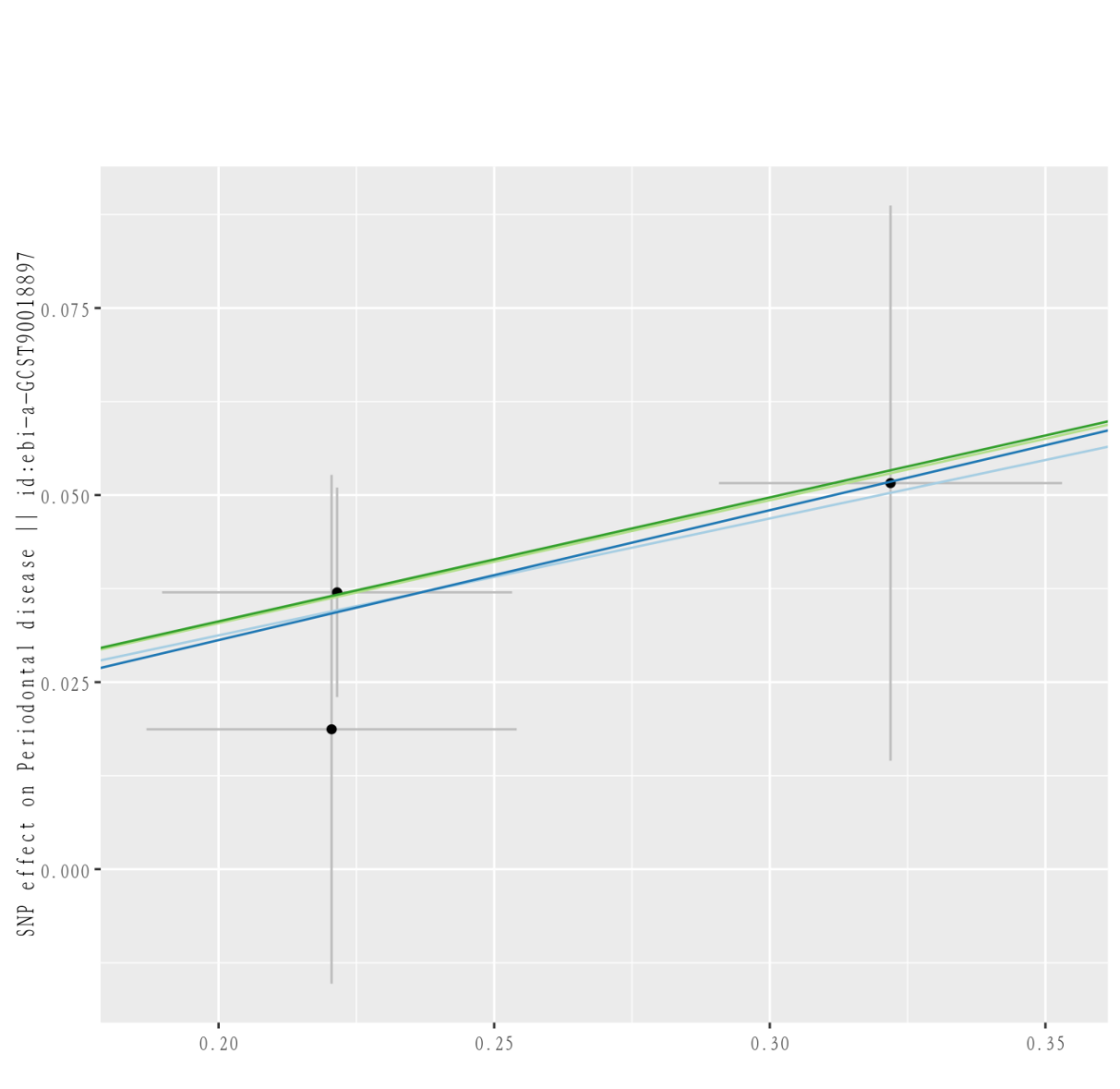


CD4 on Effector Memory CD4+ T cell


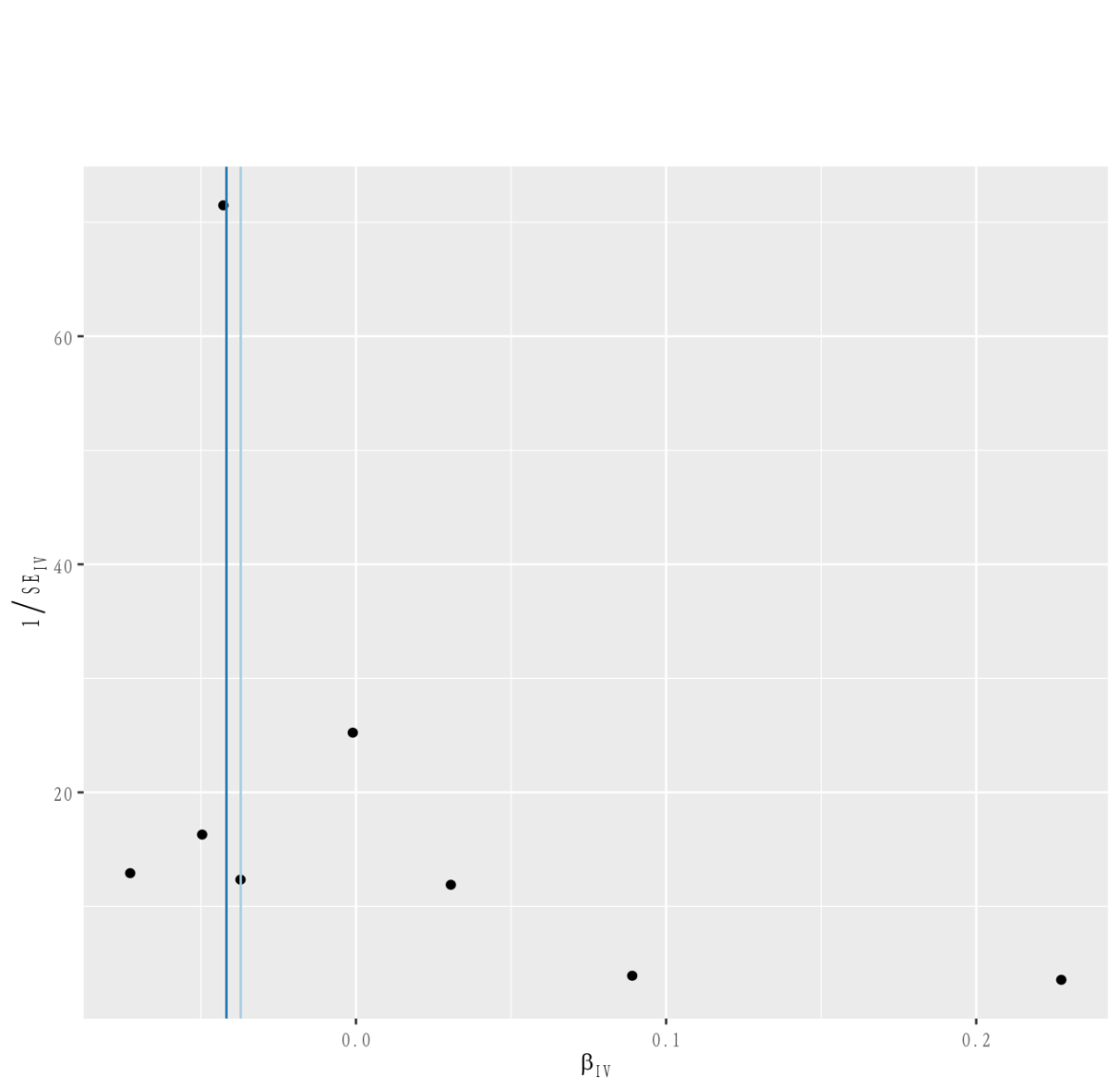

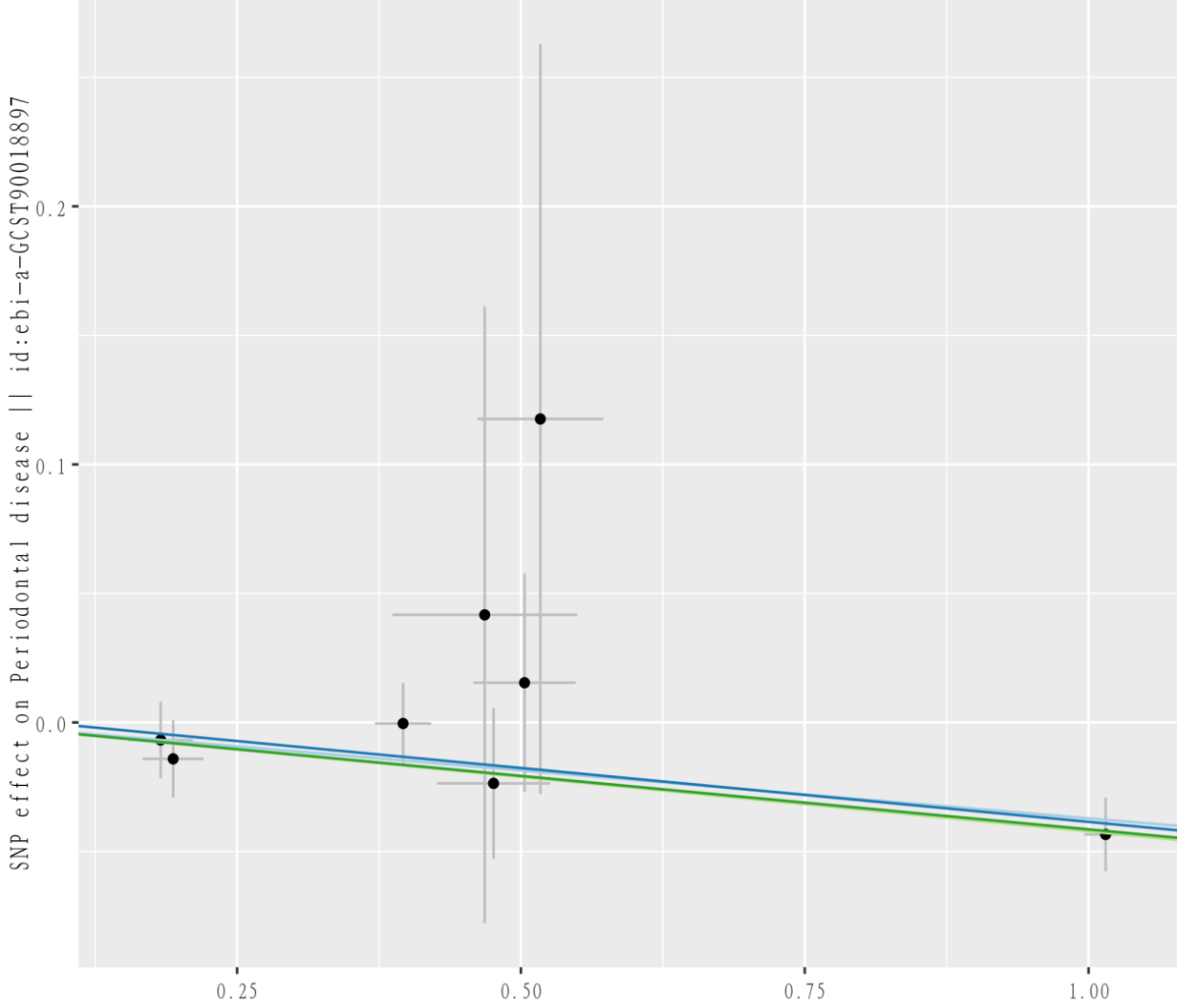


CD39+ secreting CD4 regulatory T cell


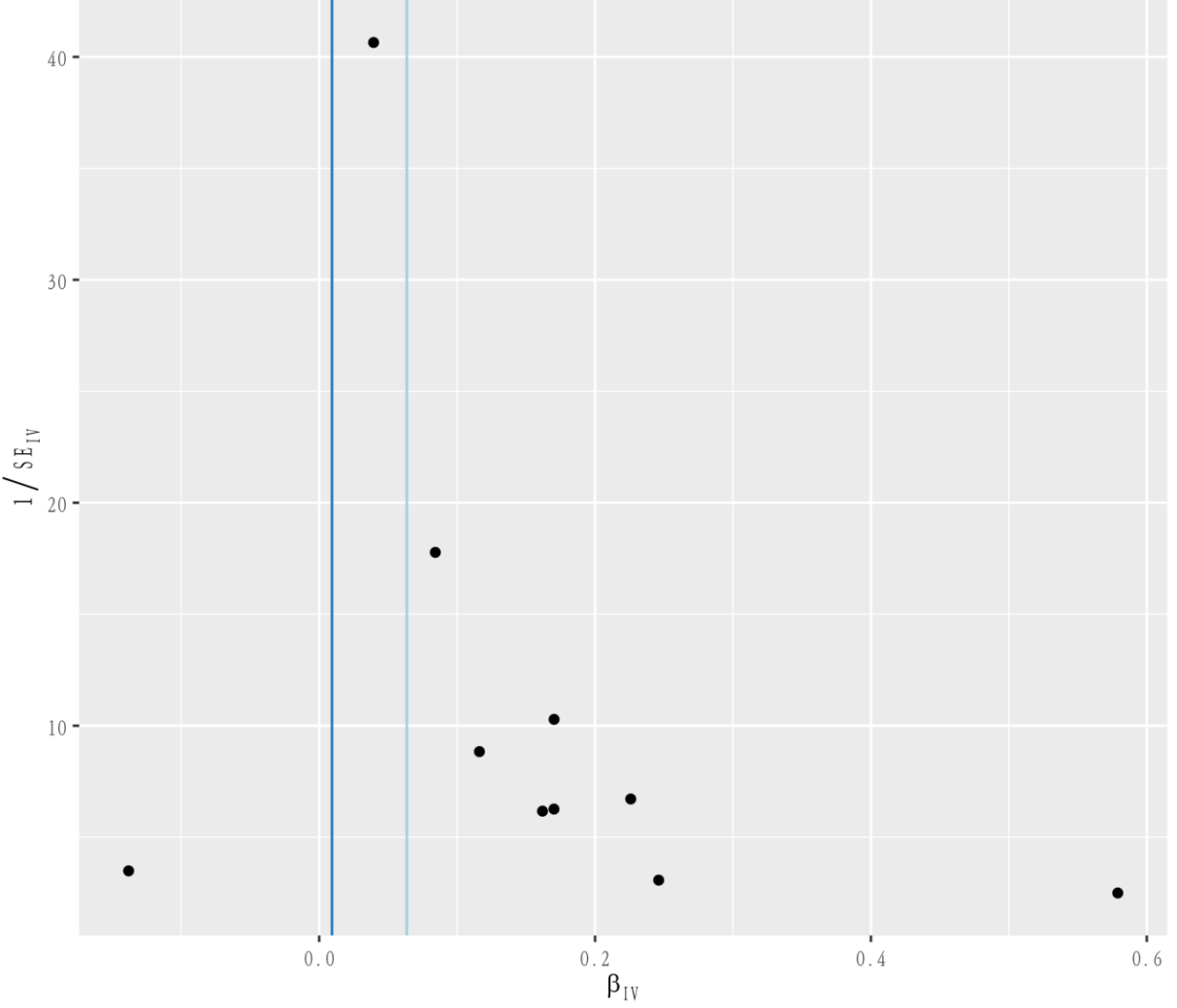

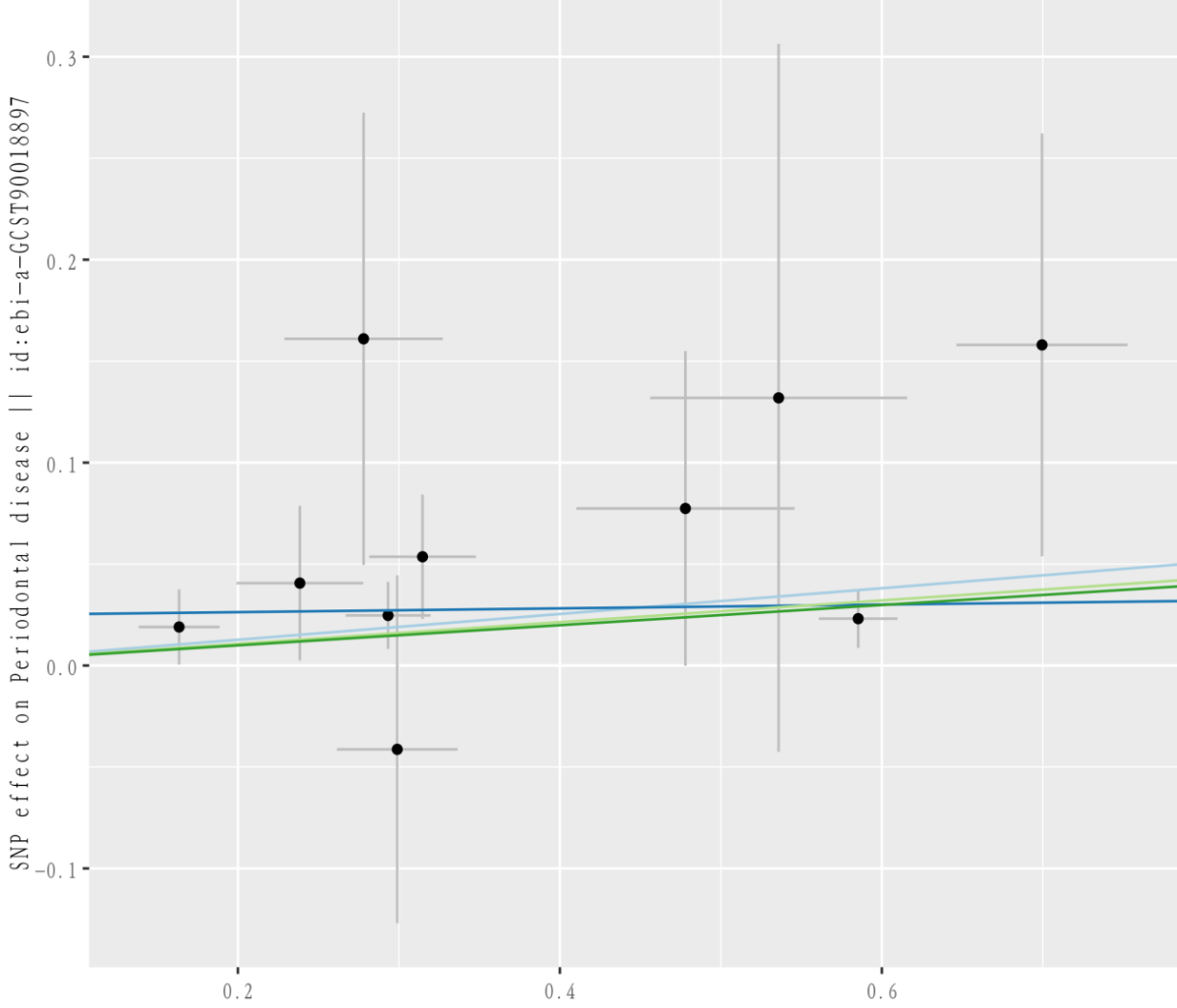


CD27 on switched memory B cell


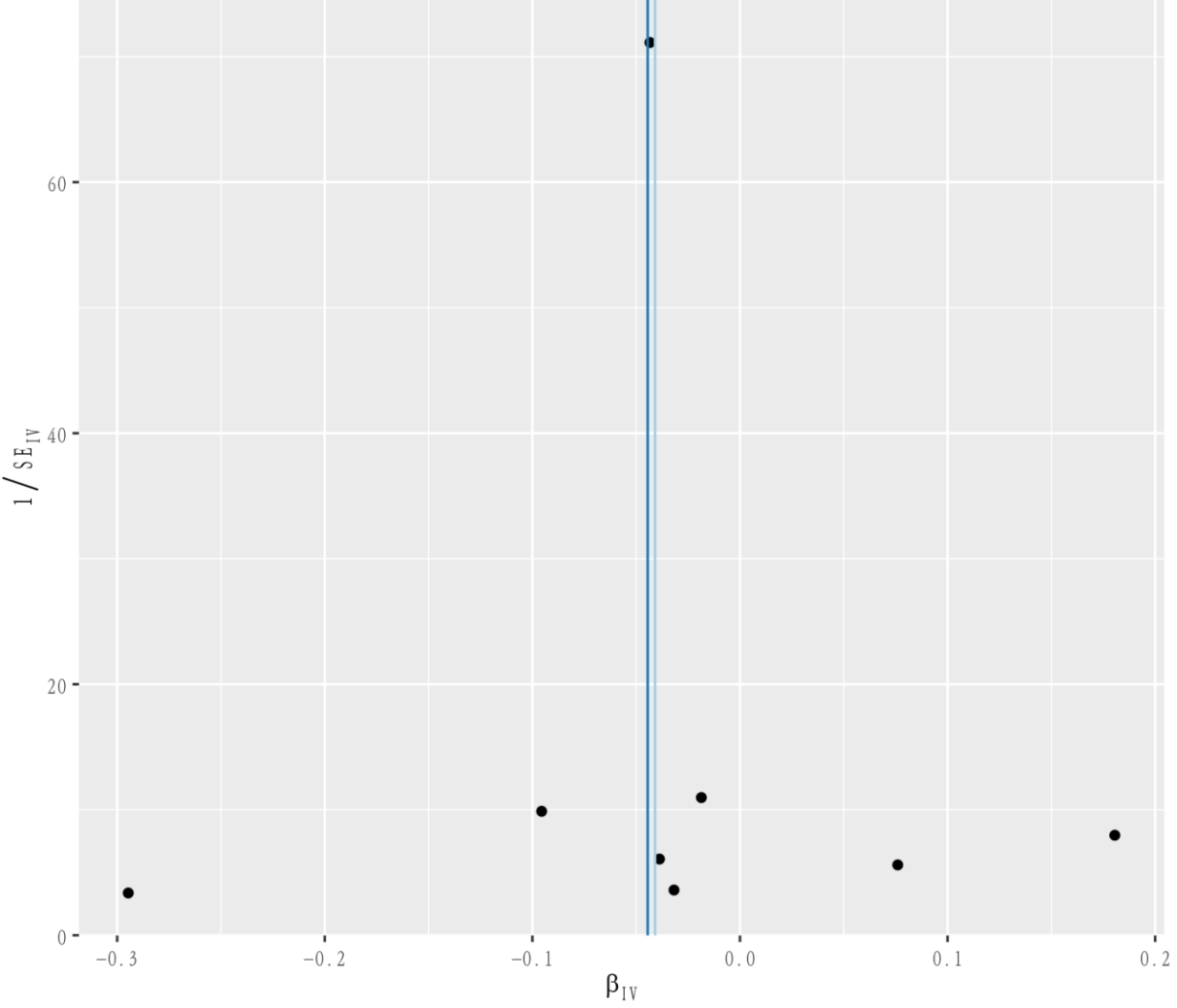

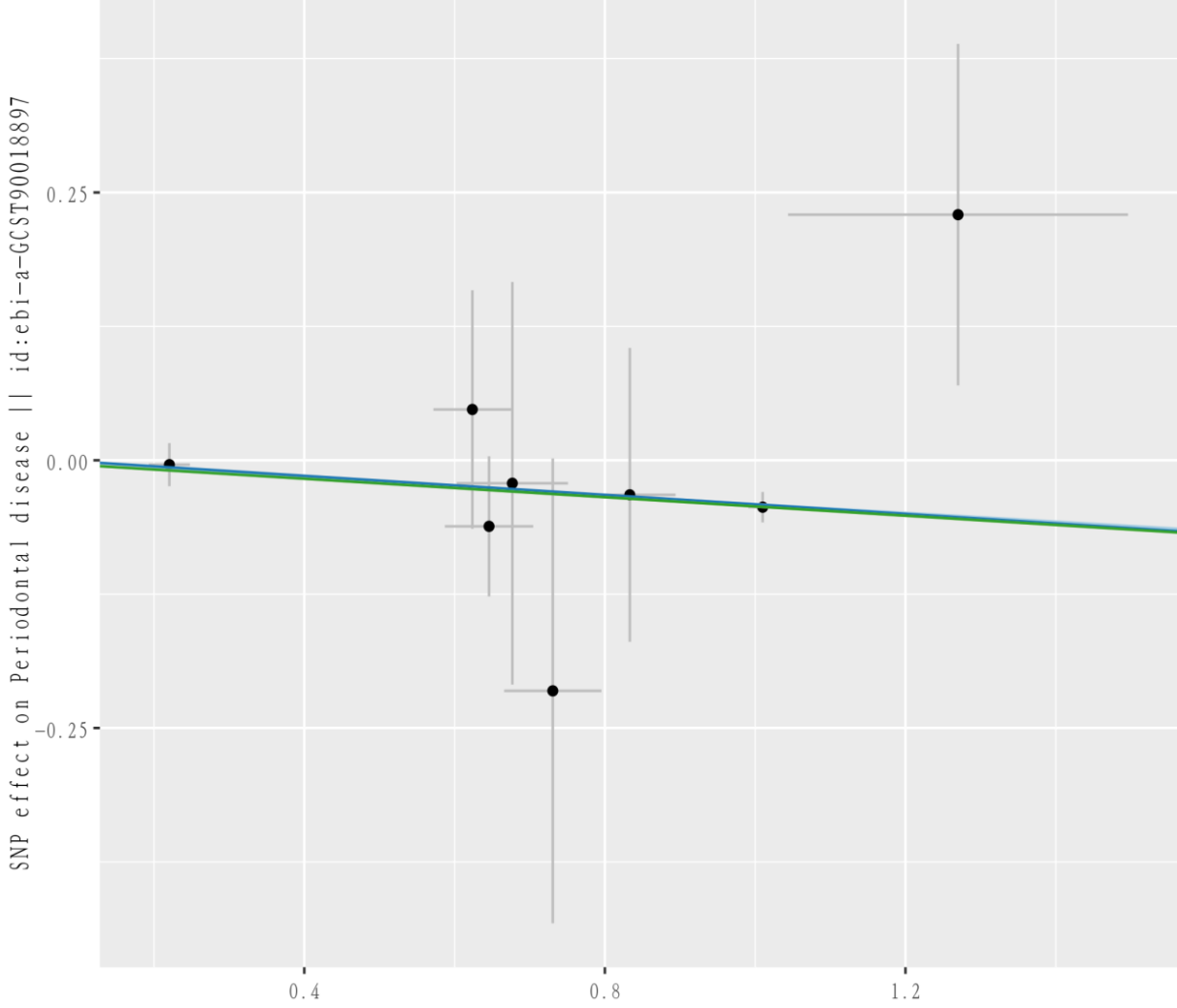


CD39+ CD8+ T cell


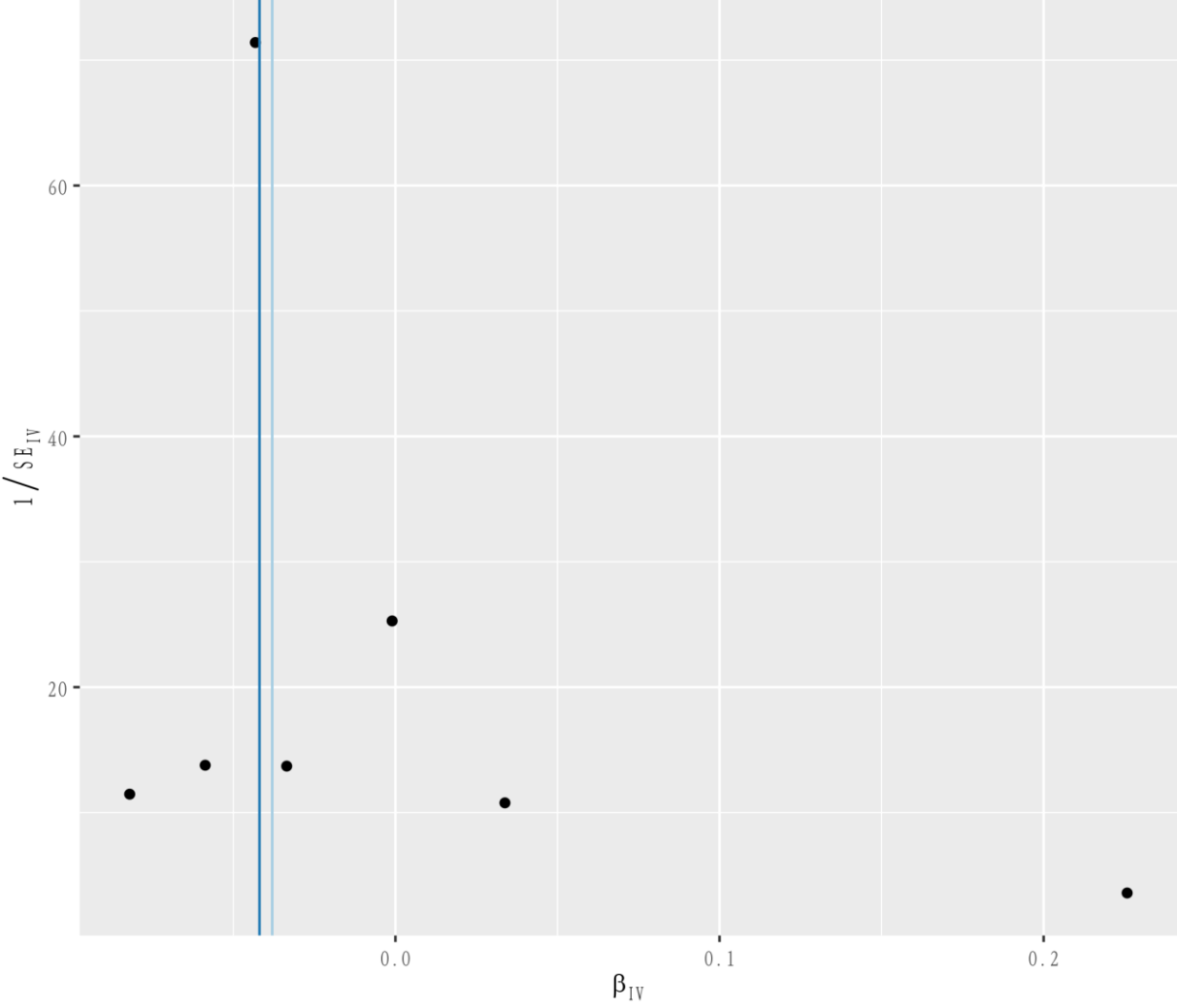

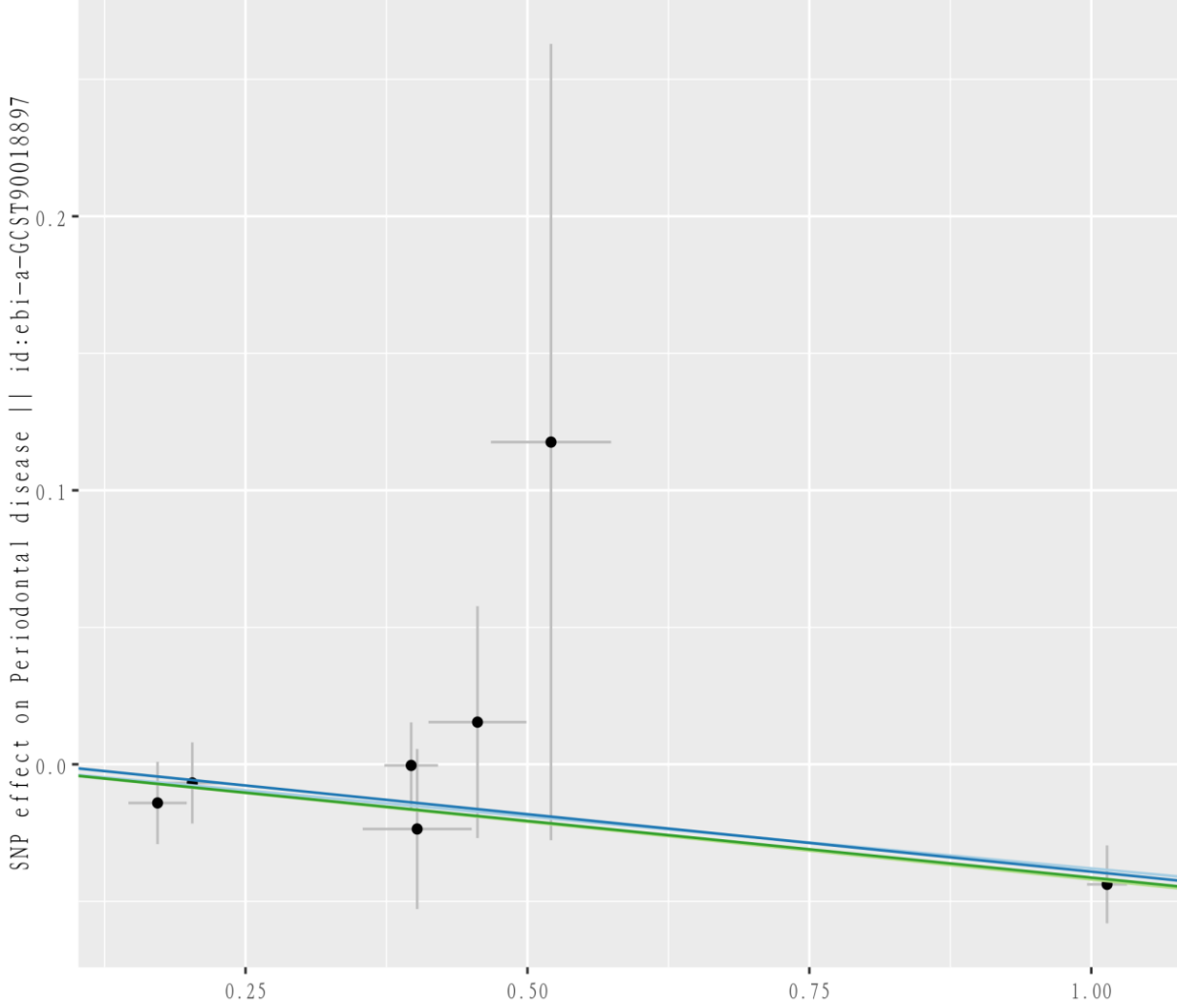


CD39+ CD4+ T cell


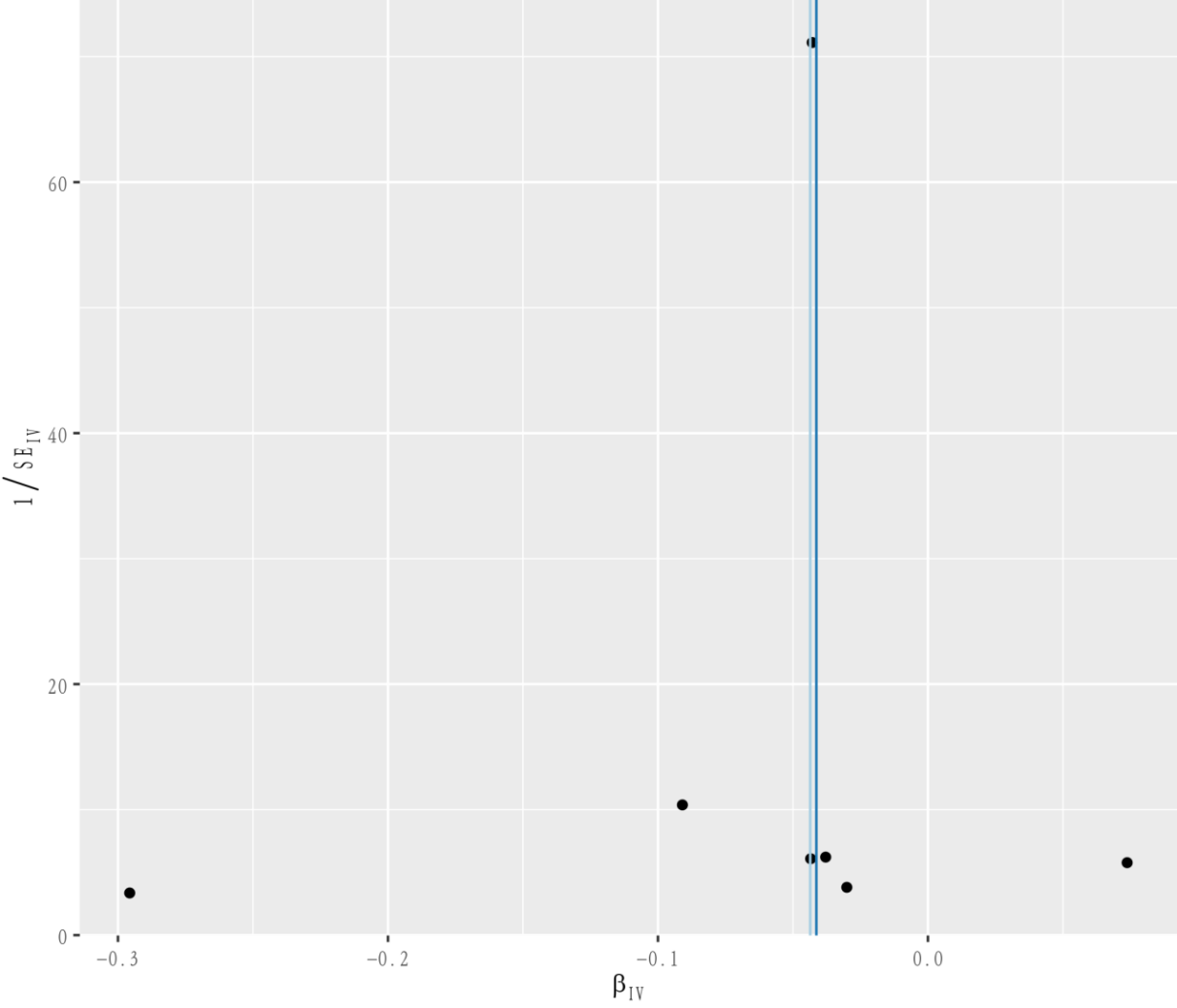

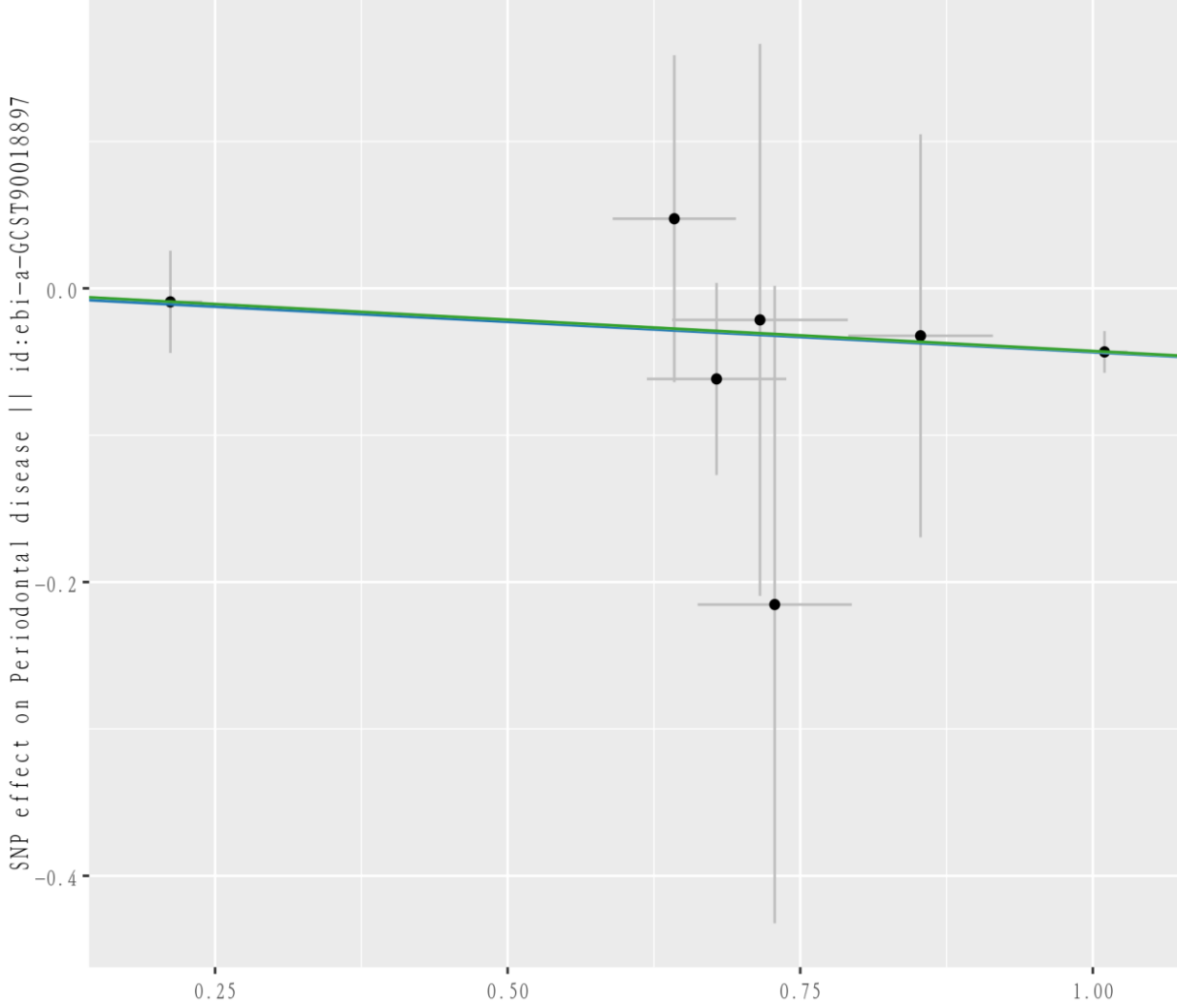


CD39+ CD8+ T cell Absolute Count
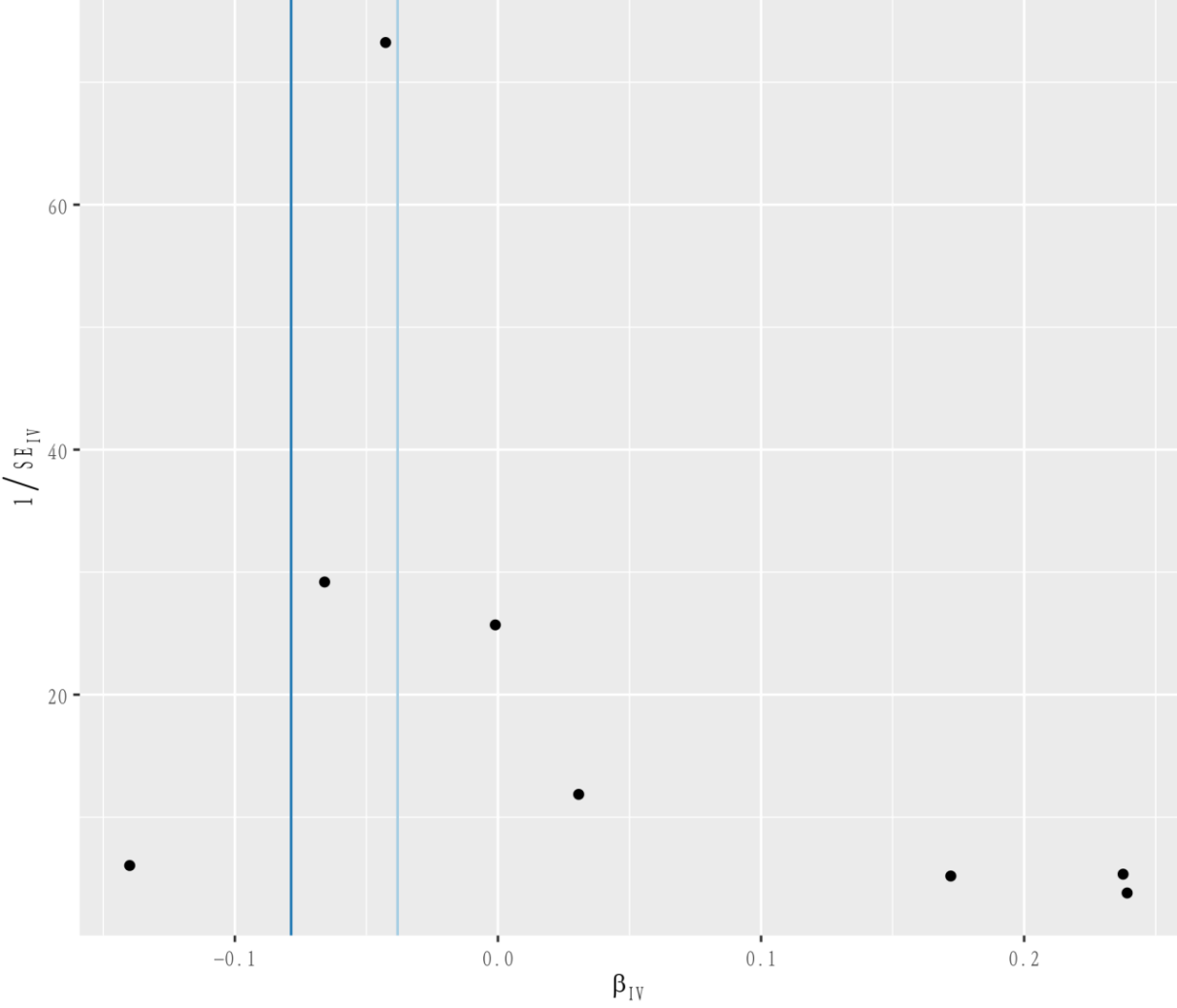

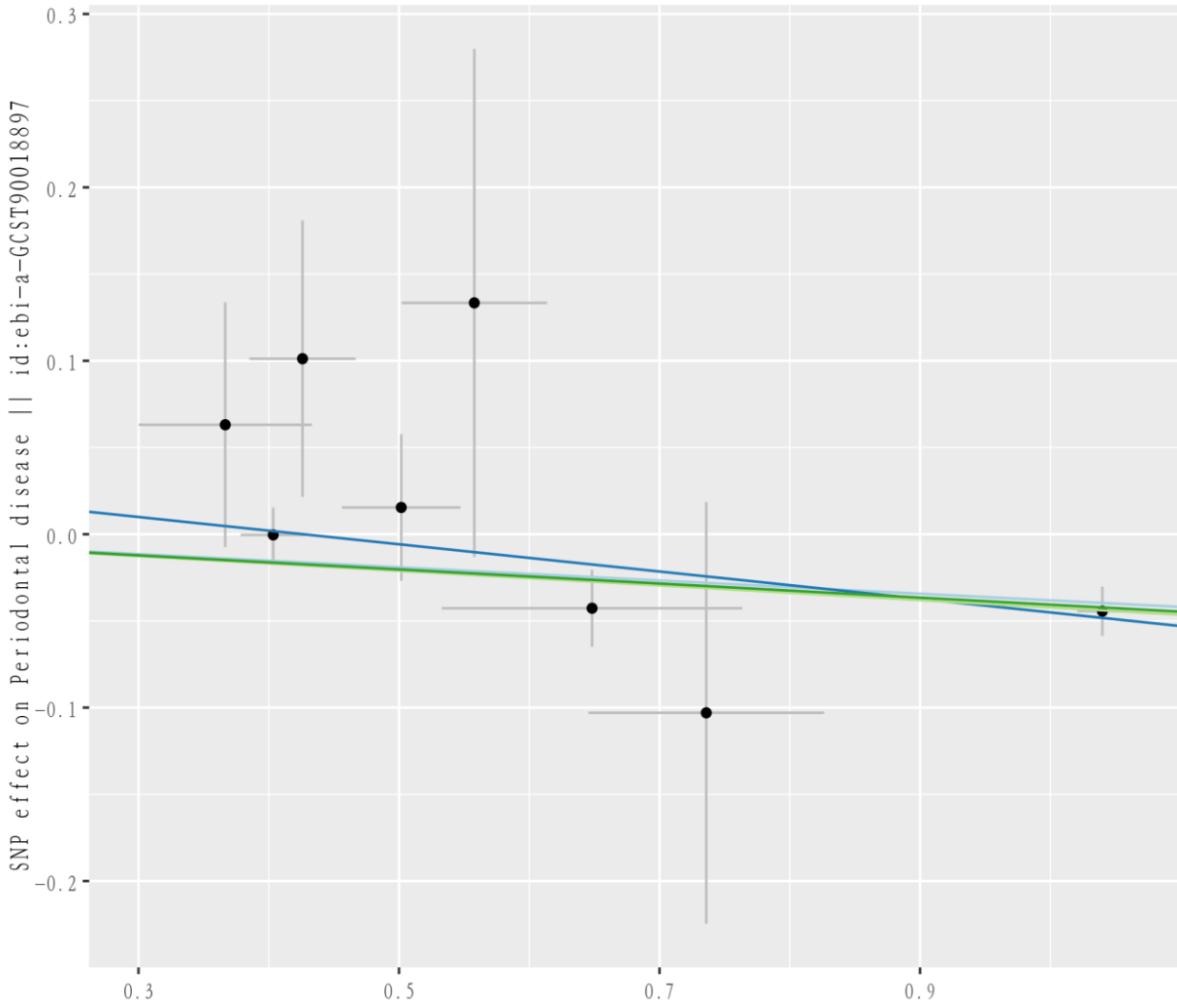


CD39+ secreting CD4 regulatory T cell
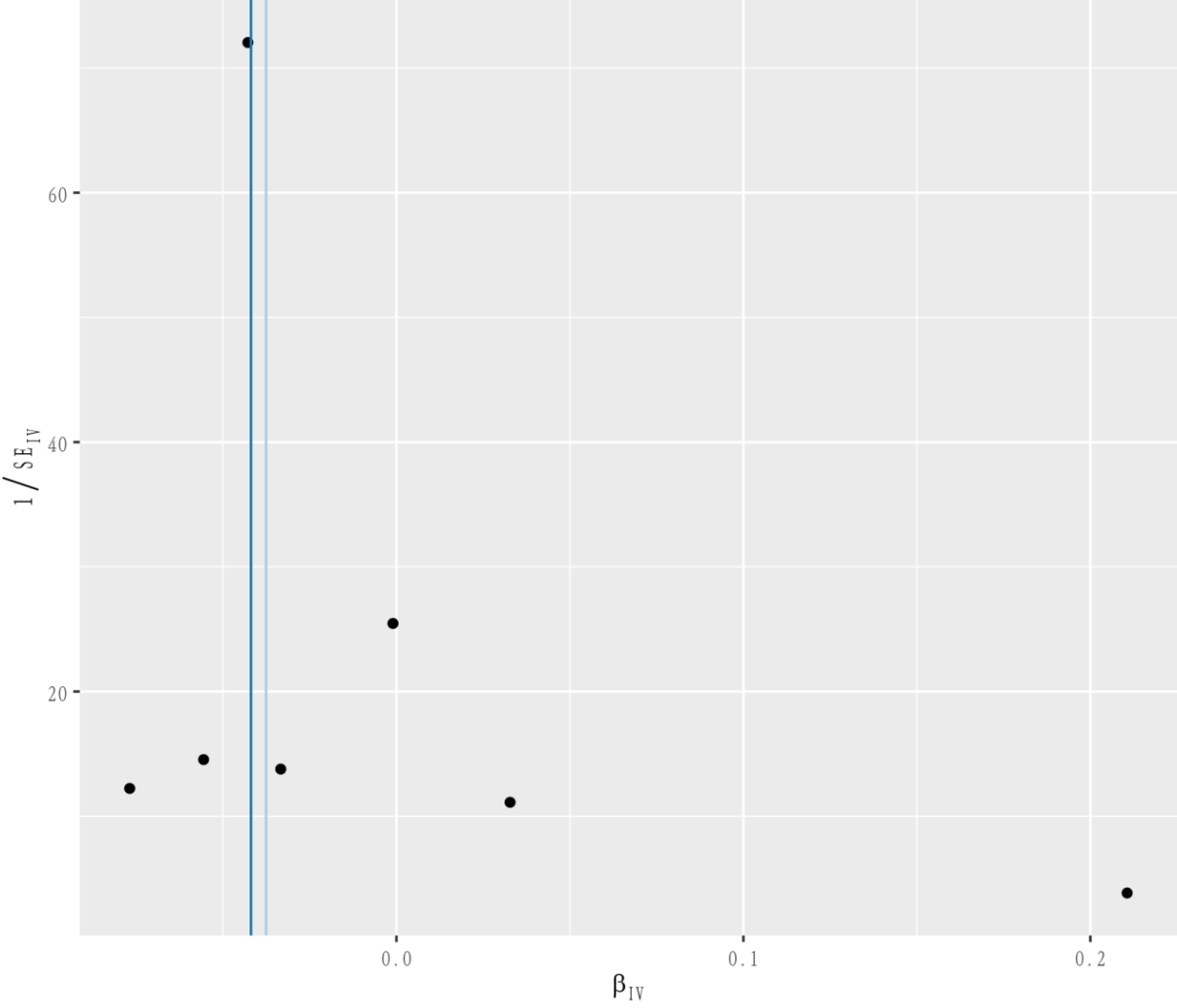

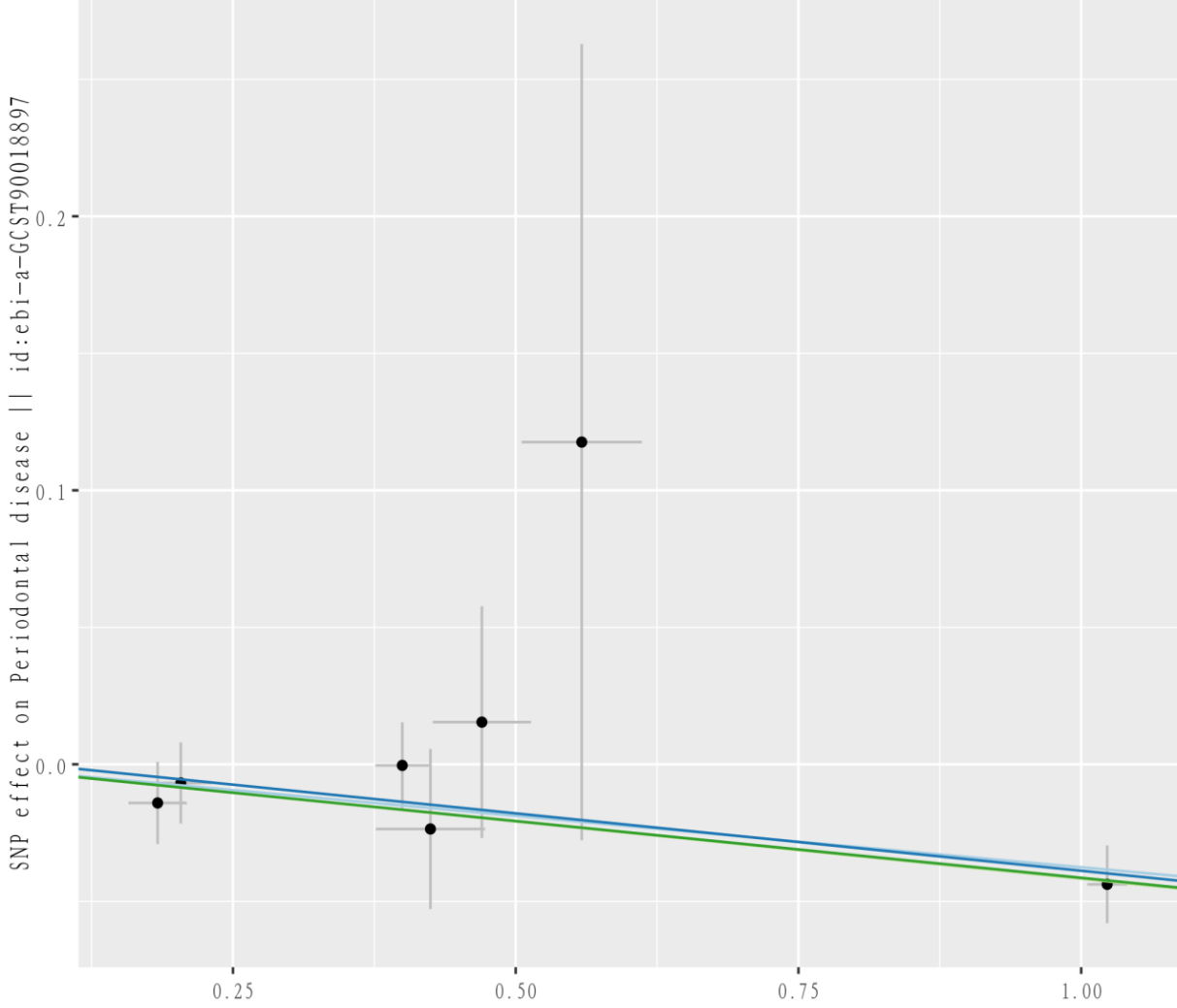


CD39+ CD4+ T cell
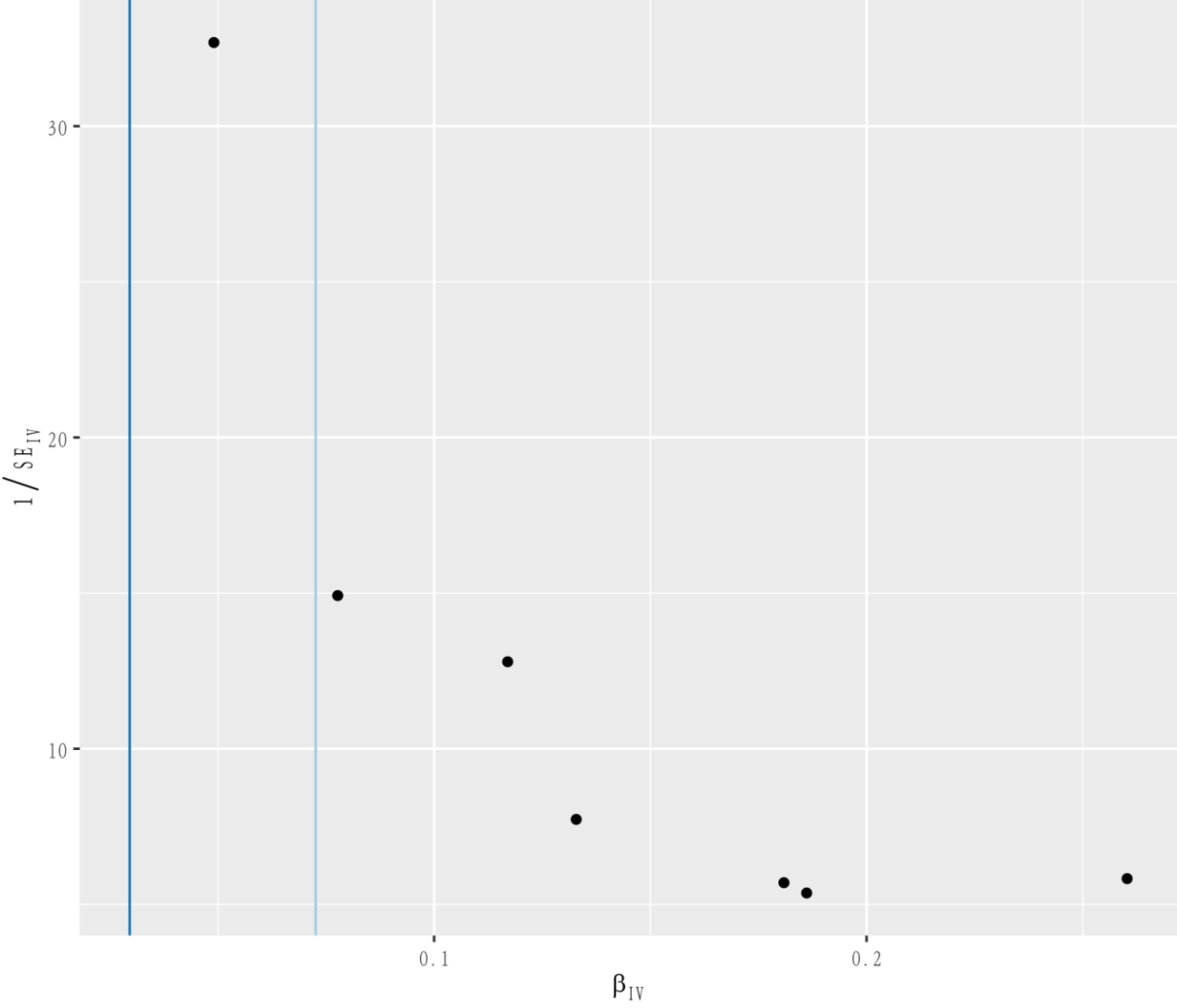

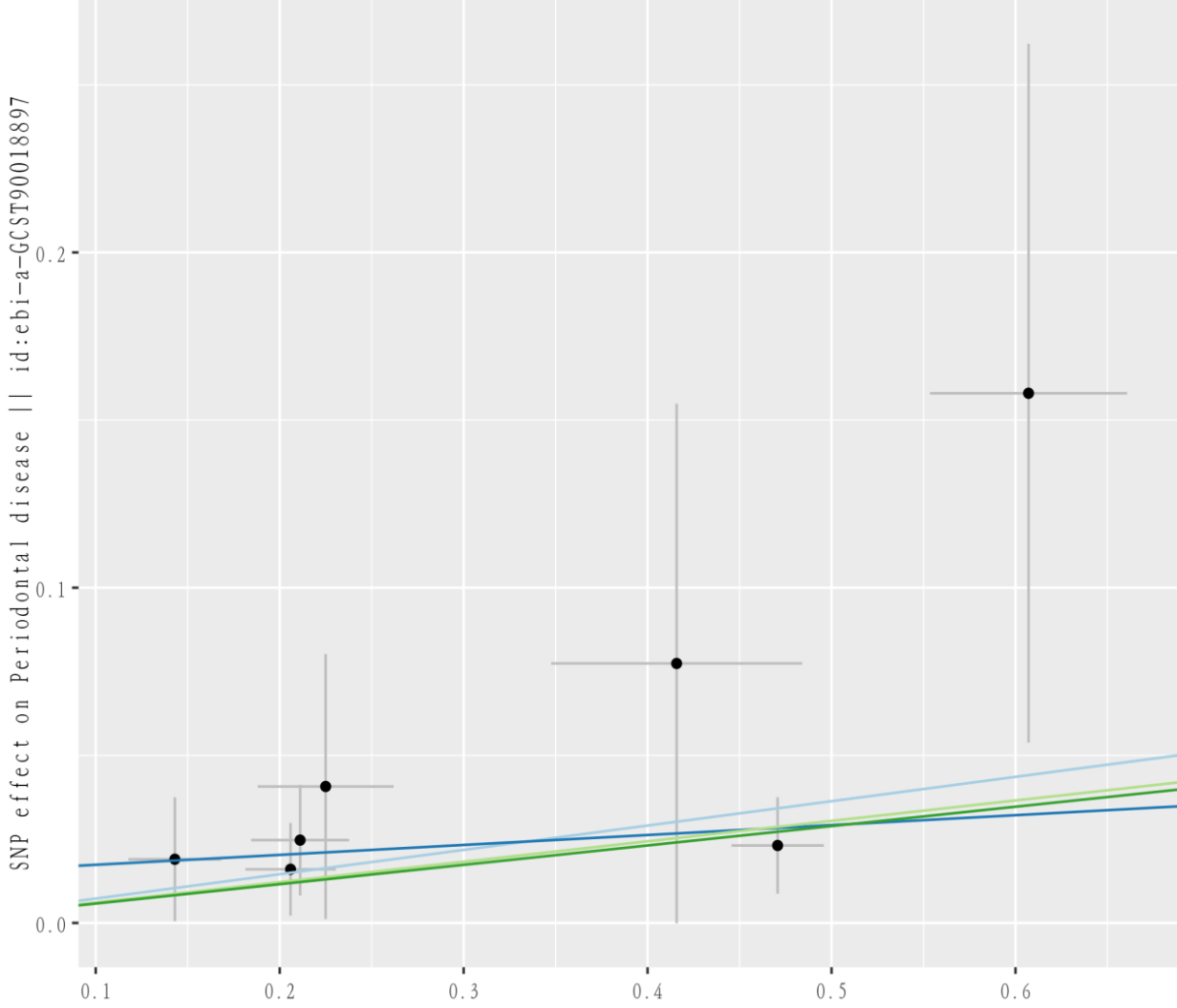


CD27 on IgD- CD38- B cell
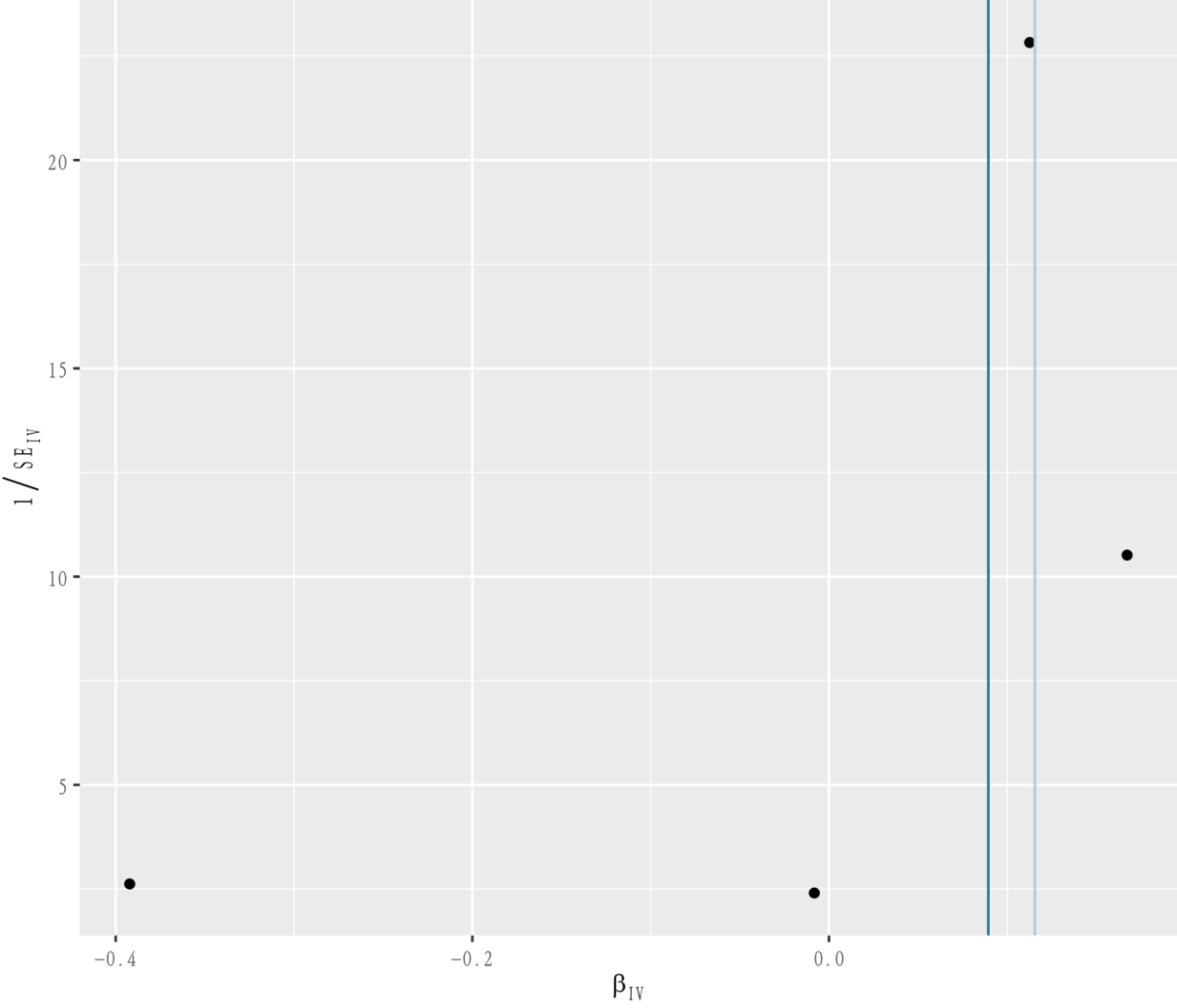

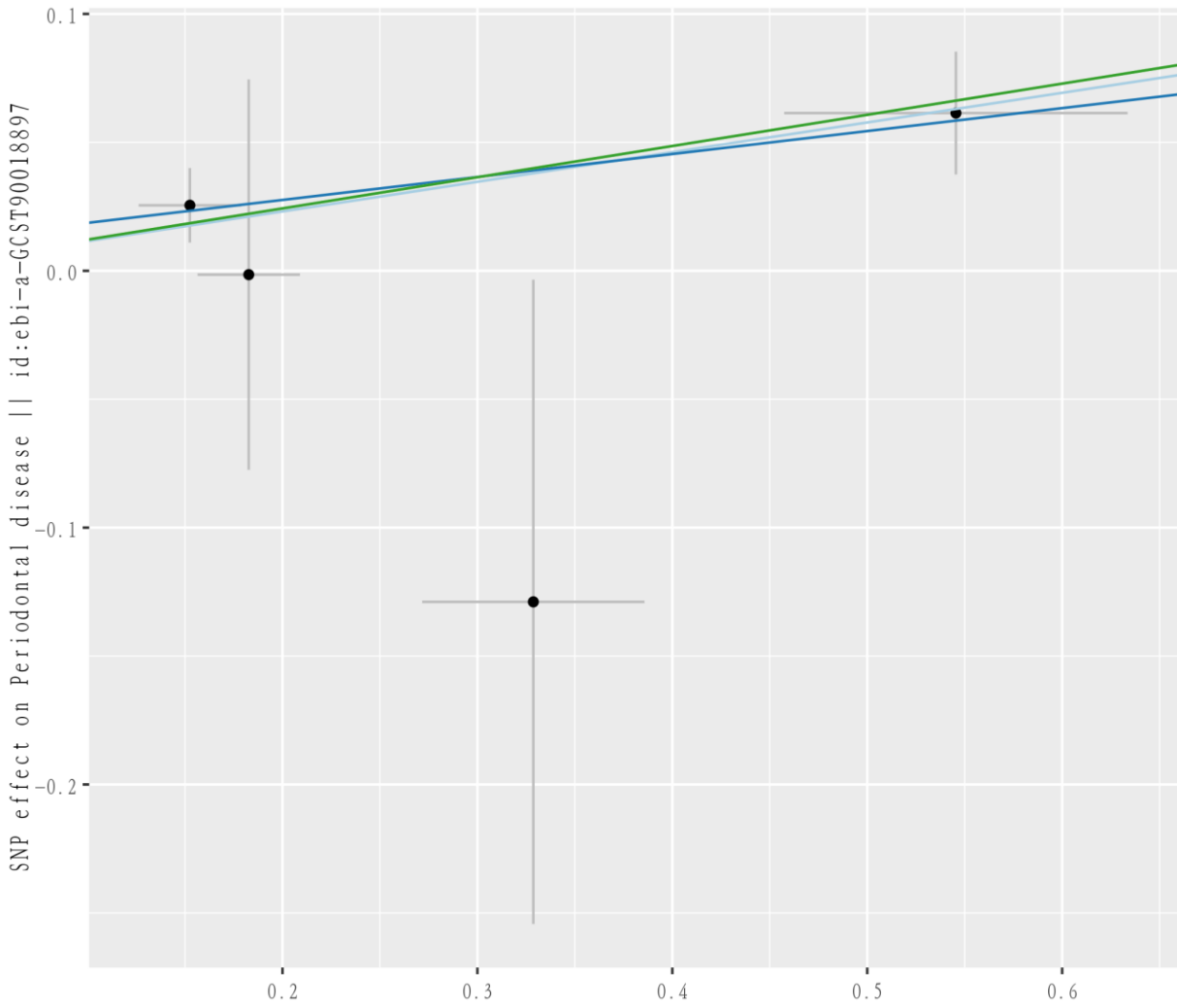


Unswitched memory B cell


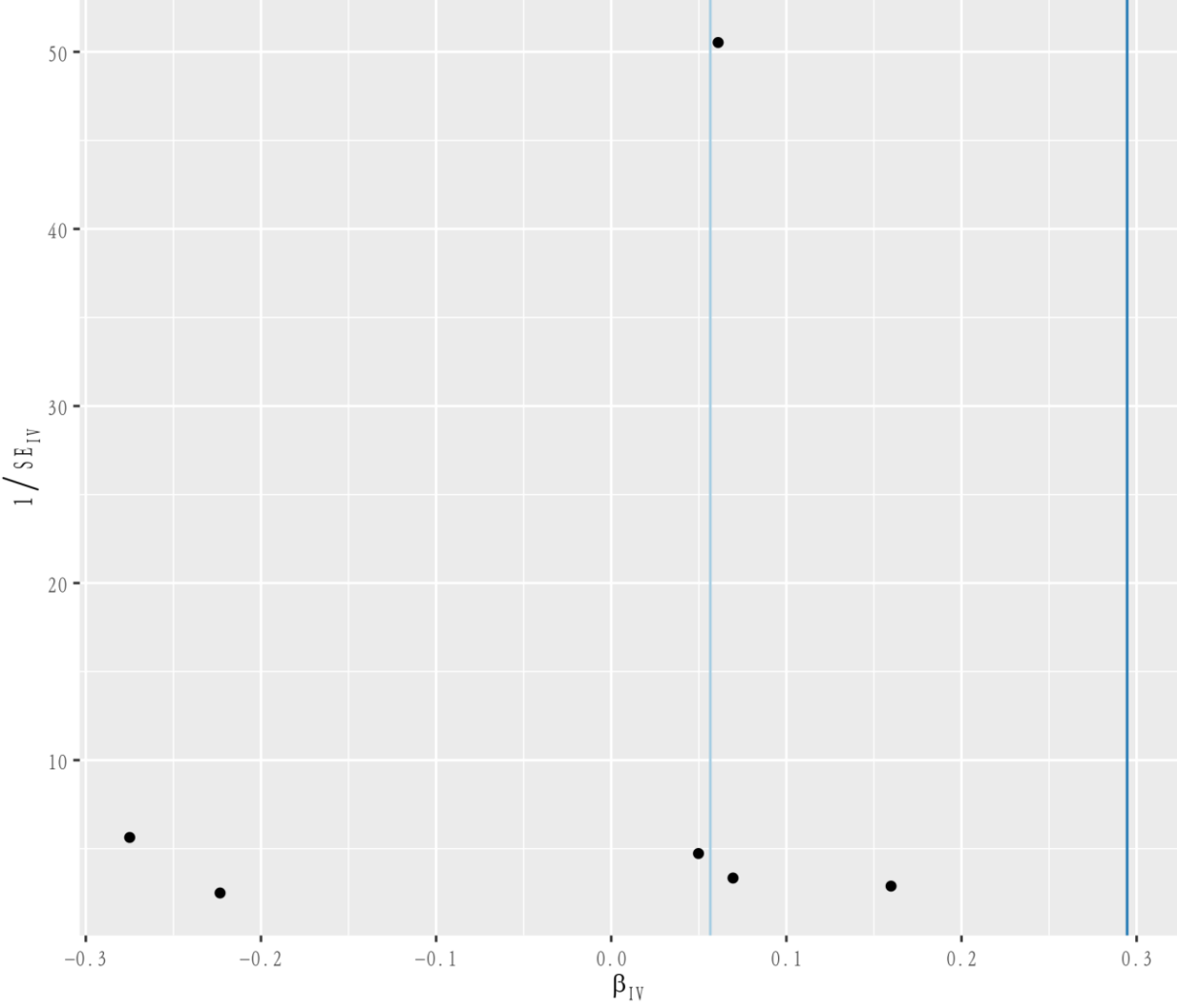


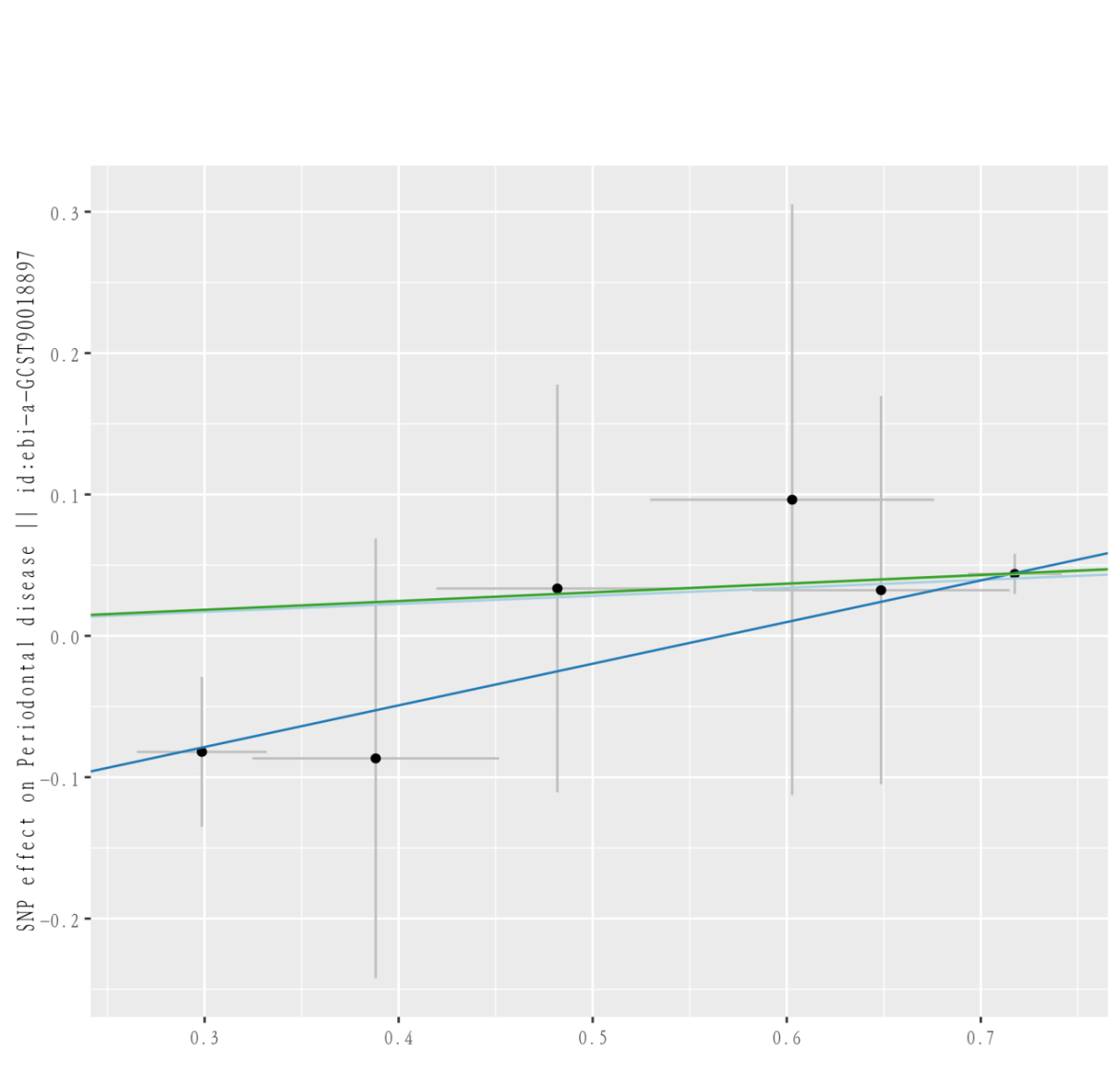


CD25 on CD39+ CD4+ T cell
